# Solid Organ Transplants Caused by COVID-19 Infection and the Outcome of Transplantation Post-COVID-19: A Systematic Review

**DOI:** 10.3390/biomedicines13020428

**Published:** 2025-02-10

**Authors:** Shadi Mahmoud, Aparajita Sarkar, Latifa AlMahmoud, Sushanth Alladaboina, Leena F. Syed, Mohammad Yaghmour, Safaa Elmoh, Meera AlShebani, Kareem Aly, Haya Al-Ansari, Mohammed Al-Mohamedi, Lina Yagan, Dalia Zakaria

**Affiliations:** 1 Department of Medical Education, Weill Cornell Medicine-Qatar, Doha 24144, Qatar; 2 Department of Medicine, University of Pennsylvania, Philadelphia, PA 19104, USA; 3 Department of Pre-Medical Education, Weill Cornell Medicine-Qatar, Doha 24144, Qatar

**Keywords:** COVID-19, SARS-CoV-2, post-COVID-19 sequelae, organ transplant, lung, liver, heart, kidney

## Abstract

The Severe Acute Respiratory Syndrome Coronavirus 2 (SARS-CoV-2) pandemic has imposed several medical and economic challenges since its onset in 2019. This is due to its ability to target the respiratory system as well as other organs, resulting in significant impacts and necessitating organ transplants. Our goal is to compile information from the existing literature to investigate how COVID-19 affects outcomes following organ transplantation. A comprehensive literature search was conducted to target studies reporting post-COVID-19 complications. We included 45 studies reporting data related to solid organ transplants, where either the recipient, organ, or donor was affected by SARS-CoV-2. The majority of the included studies concluded that organ transplantation following COVID-19 infection could be performed safely and with similar outcomes to non-COVID-19 patients, regardless of whether the organ, donor, or recipient was affected by COVID-19. No deviation from standard immunosuppression regimens or surgical protocols was necessary either, further re-assuring the feasibility of these transplants as viable treatment options. This applies to organ transplants involving the lungs, kidneys, liver, or heart. However, there was a limited number of studies in some areas, which warrants the need for additional research in order to reach more concrete conclusions pertaining to COVID-19’s effect on organ transplants.

## 1. Introduction

Over the past two decades, coronaviruses have emerged as a significant threat on multiple levels due to their high mortality rate and severe disease states, prompting calls for accelerated development of vaccines and stricter changes to guidelines and healthcare protocol. The rapid spread of Severe Acute Respiratory Syndrome Coronavirus (SARS) in 2003, followed by Middle East respiratory syndrome (MERS) in 2012, and the ongoing COVID-19 pandemic since 2019 created several challenges that required an immediate global response due to their relatively high rapid spread and case fatality rates. SARS-CoV, MERS-CoV, and Severe Acute Respiratory Syndrome Coronavirus 2 (SARS-CoV-2) were the viruses responsible for the aforementioned challenges, respectively, with the current pandemic proving to be the most challenging on both a social and economic level [1]. Classified as a respiratory pathogen, Coronavirus Disease 2019 (COVID-19) has been reported to cause a wide variety of symptoms both in terms of severity and chronicity. In terms of short-term effects, it can impact several systems and lead to symptoms such as fever, cough, shortness of breath (SOB), pneumonia, palpitations, acute myocardial infarction (MI), stroke, and several other sequelae [2]. Long-term effects, also known as post-acute sequelae of SARS-CoV-2 (PASC), may arise in essentially any organ system and include lung fibrosis, myocarditis, memory issues, chronic kidney disease (CKD), inflammatory bowel syndrome (IBS), and psychological symptoms such as post-traumatic stress disorder (PTSD) [3]. The frequency with which PACS were seen led to the World Health Organization [4] (WHO) terming them as “long COVID” (LC), which is given to patients experiencing symptoms that persist or return 3 months following infection with COVID-19.

### 1.1. COVID-19 Infection May Cause Organ Damage Requiring Organ Transplants

**Figure 1:**
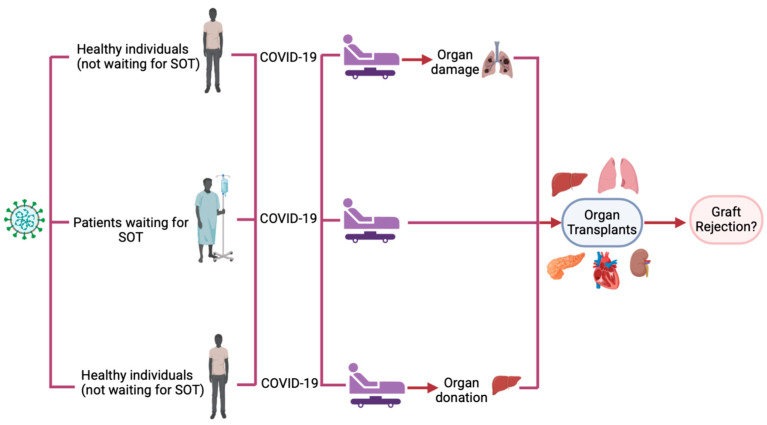
The study structure involved 3 categories of patients with a history of COVID-19 including healthy individuals who developed organ damage post-infection requiring organ transplantation, patients who contracted COVID-19 while waiting for organ transplantation, and healthy individuals who donated organs post-COVID-19 infection. SOT: solid organ transplants.

As previously mentioned, infection with COVID-19 can lead to major injury to any organ system. Some reported examples of this include acute respiratory distress syndrome (ARDS), lung fibrosis, myocarditis, arrhythmias, heart failure, acute kidney injury (AKI), CKD, hepatitis, liver failure, and many more [5]. The pathophysiology behind COVID-19 causing organ injury has been proposed by several studies. One of the most commonly reported mechanisms is the use of spikes membrane protein and host protease TMPRSS2, which allows the virus to target areas with ACE-2 receptor expression [6]. This leads to the observed changes seen in the lungs, liver, and many other organs. However, studies emphasize that this is only a part of why the virus leads to organ injury, and it is, in fact, the immune system that triggers such detrimental changes. This is due to the hyperimmune response exhibited through a “cytokine release storm”, which leads to a pro-inflammatory state with an imbalanced coagulation cascade [6,7]. This results in increased mortality through MI, stroke, pulmonary embolism (PE), deep venous thrombosis (DVT), and several other complications. For this reason, the immune response following COVID-19 infection should be considered a primary factor in proposed mechanisms of COVID-19-induced organ damage. COVID-19-induced organ damage may require organ transplantation. However, it is important to understand whether the immune dysregulation associated with the infection may affect the outcome of transplantation post-infection or not. Moreover, this immune dysregulation may also impact any post-COVID-19 transplants, including patients who contracted COVID-19 while waiting for transplantation and patients who donated organs post-COVID-19 infection. Therefore, this study investigates the outcome of solid organ transplants in the following three categories: patients who underwent transplantation due to organ damage caused by COVID-19, patients who contracted COVID-19 infection while waiting for transplantation, and patients who received an organ from a donor previously infected with COVID-19. Figure 1 illustrates the categories of patients included in this study.

### 1.2. Immune Dysregulation and the Outcome of Transplanation Post-COVID-19 Infection

COVID-19 infection has a significant impact and influence on the immune profiles of patients during the convalescence period. The most striking is the persistence of lymphopenia at least 4 months after discharge [8]. Several studies have noted the presence of inflammatory monocytes and alterations in the lymphocyte populations [9]. It is important to determine the impact of such profiles on the outcome of solid organ transplants occurring post-COVID-19 infections. The differences in immune profiles between healthy controls and patients with mild, moderate, and severe disease were investigated. Patients who had severe disease had a significant increase in the frequency of monocytes (compared to controls) and NK cells (compared to mild and moderate disease) 1 to 3 months after recovery. A higher level of polymorphonuclear myeloid-derived suppressor cells was also seen in all patients (mild, moderate, and severe) compared to controls. Increased levels of innate immune cytokines (except TNFα, IFNγ, and IL4/IL-13) were seen as the severity of the disease increased. Lastly, increased levels of monocytes and cells that produce IL-6 and TNF-α were seen to be correlated with the development of long COVID symptoms [10]. Notably, patients who required solid organ transplantation post-COVID-19 infection may have had severe infection. The prolonged decrease in NK and NKT cells, coupled with increased levels of monocytes and cytokines such as IL-6 and TNF-α, may exacerbate inflammation and compromise immune regulation, posing challenges for transplant recipients by increasing the risk of graft rejection or delayed recovery. This may apply to solid organ transplants post-COVID-19 infections regardless of the stem cause of organ damage requiring transplantation, i.e., whether COVID-19 was the cause of damage or not.

Our study focuses on the following categories that might be affected by patients’ dysregulated immune profiles after the recovery from COVID-19.

#### 1.2.1. The Outcome of Solid Organ Transplants in Patients Who Had Organ Damage Caused by COVID-19

The majority of these patients had severe COVID-19 leading to organ damage, which might have been caused by direct viral attack and/or immune dysregulation. As explained above, this immune dysregulation may last after recovering from the infection. Patients under this category could be considered as long COVID patients by definition. This is because their post-COVID-19 complications never resolved within 3 months post-infection without organ transplantation. How may the dysregulated immune profile of the recipient post-COVID-19 infection impact the immune response against the graft?

#### 1.2.2. The Outcome of Solid Organ Transplants in Patients Who Contracted COVID-19 During the Waiting Period

A patient population of interest includes those who were waitlisted for an organ transplant following severe non-COVID-19 organ damage and subsequently contracted COVID-19 during the waiting period prior to the procedure. Exploring this point may prove significantly beneficial as it may answer questions regarding the potentially detrimental immunological effect COVID-19 may have on transplant outcomes in waitlisted patients. Some of these effects may manifest as graft rejection, post-transplant infections, and other complications that render post-operative patient management increasingly challenging. Information on the immune profile of patients, who ultimately developed graft rejection post-transplant during infection with COVID-19 may help us understand the factors contributing to the immune mechanism behind graft rejection. Just like the previous category, the immune dysregulation post-COVID-19 infection may last after recovering from the infection. How may the dysregulated immune profile post-COVID-19 infection of the recipient impact the immune response against the graft even if COVID-19 did not cause organ damage?

#### 1.2.3. The Outcome of Solid Organ Transplants in Patients Who Received Organs from Donors Who Were Previously Infected with COVID-19

The immune profile of the solid organ donors (both live and deceased) was found to have a significant impact on the outcome of organ transplants [11,12]. How may COVID-19 infection of the donor impact the immune response against the graft?

Our study aims to take a holistic approach to exploring the etiology behind COVID-19-induced organ damage, as well as whether previous COVID-19 infection would negatively impact outcomes in patients following organ transplants based on the above three categories.

## 2. Materials and Methods

The preferred reporting items for systematic reviews and metanalysis (PRISMA) statement was used to develop the protocol (registered at inplasy, registration number: INPLASY2024110125) of this systematic review [13].

### 2.1. Information Sources and Search Strategy

This study is part of a large project that investigates the long-term and severe complications of COVID-19. We conducted a comprehensive search through our institutional information professional who prioritized sensitivity to retrieve all relevant studies. The following databases were searched in October 2023: PubMed, Medline (Ovid, 1946—Current), Embase (Ovid, 1974–2021), Scopus, Web of Science, Science Direct, and Cochrane Library. The search was designed around keywords and controlled vocabulary that focused on “long COVID” and variants (see File S1 for the full search details). No language or date restrictions were used. All database search results were imported into EndNote (version 19) and exported to Covidence, where duplicates were removed prior to initial screening (File S1).

### 2.2. Eligibility Criteria

No restrictions were made based on gender, age, or country. Duplicates were removed, and any articles that were not in English or did not have primary data, such as review articles, were excluded. We excluded all conference abstracts, and studies were included only if their full articles were published in peer-reviewed journals. During the full-text screening, any studies that reported a solid organ transplant following COVID-19 infection were included. This applied to solid organ transplants conducted for recipients who previously had COVID-19 whether the organ damage was caused by COVID-19 or not. Furthermore, we also included any studies that reported a solid organ transplant when the donor previously had COVID-19.

### 2.3. Study Selection and Data Collection

Title and abstract screening and full-text screening were conducted by two independent reviewers for each study using Covidence, and disagreements were resolved by consensus. Data were extracted and crosschecked by two independent reviewers.

### 2.4. Data Items

Demographic and clinical data, including age, sex, comorbidities, treatments, and outcomes, were collected. Continuous variables were expressed as mean ± standard deviation or range of results. Categorical variables were expressed as percentages.

### 2.5. Risk of Bias and Quality Assessment

Different quality assessment methods were used depending on the type of study. The Newcastle–Ottawa Quality Assessment Scale was used to assess the cohort studies (NOS) [14]. The scale developed by Murad et al. [15] was used to assess the case reports and case series. Quality assessment was conducted and crosschecked by two independent reviewers.

### 2.6. Data Analysis

Solid transplants were categorized in our study into three main categories: the recipient previously had COVID-19 and the organ damage was not caused by COVID-19; the donor previously had COVID-19; and the recipient previously had COVID-19 and the organ damage was caused by COVID-19. The latter was consequently classified based on the type of organ, which included the lung, liver, and heart.

## 3. Results

**Figure 2:**
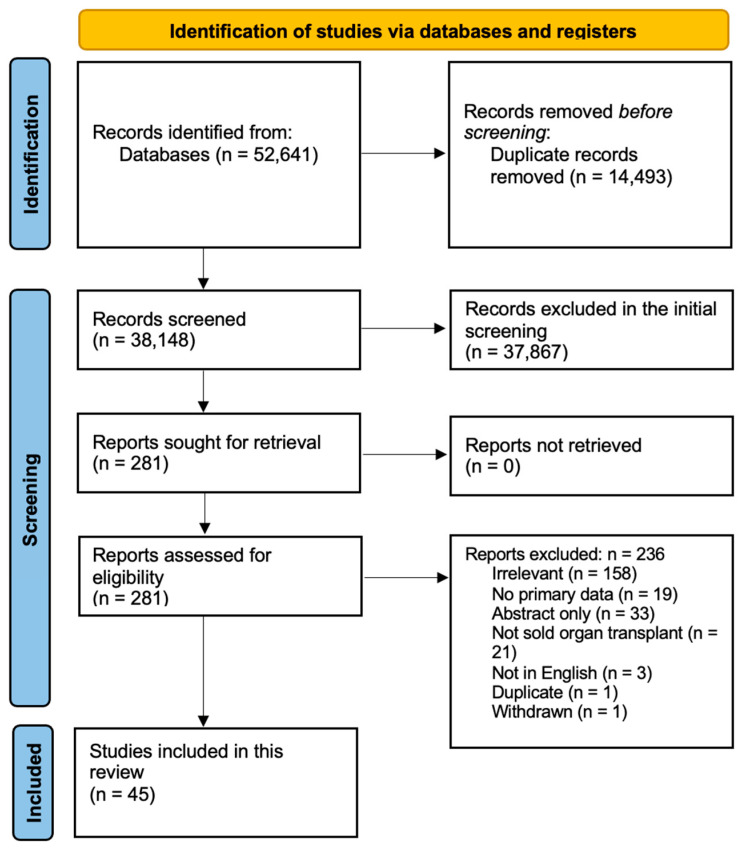
Protocol of database search, screening, and study selection.

Figure 2 shows a flow diagram of our protocol. After removing the duplicates, the titles and abstracts of 38,148 studies were screened, of which 281 were selected for full-text screening. Only 45 studies met our inclusion criteria. Of the 236 excluded studies, 158 were irrelevant, 19 lacked primary data, 33 were conference abstracts, 21 did not report solid organ transplants, 3 were not in English, 1 was withdrawn, and 1 was a duplicate. Supplementary Table S1 summarizes the demographic and clinical data of the included subjects as well as the quality assessment scores for each study [16,17,18,19,20,21,22,23,24,25,26,27,28,29,30,31,32,33,34,35,36,37,38,39,40,41,42,43,44,45,46,47,48,49,50,51,52,53,54,55,56,57,58,59,60].

### 3.1. Types of Studies and Demographic Data

Of the 45 included studies, 26 were case reports, 11 were case series, 8 were cohort studies, and 1 was an observational autopsy study. Among the 45 included studies, there were 18 from the USA, 5 from India, 1 from Germany, 4 from Austria, 2 from Turkey, 2 from Italy, 1 from Israel, 3 from Brazil, 3 from Japan, 1 from Croatia, 1 from Australia, 1 from Romania, 1 from Canada, 1 from Sweden, and 1 from France.

**Figure 3:**
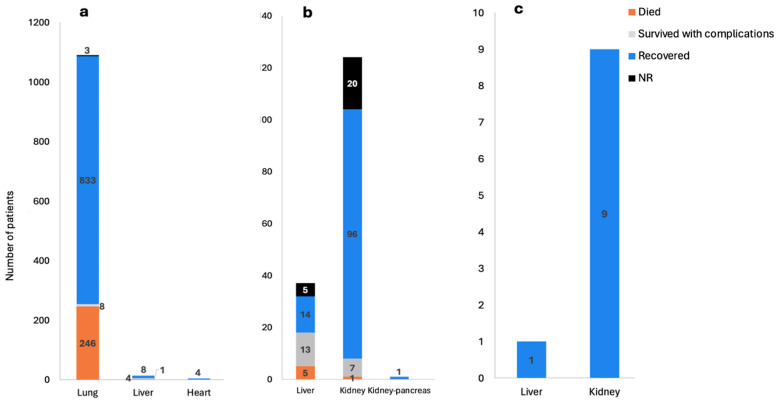
Categories of reported solid organ transplants post-COVID-19 and the outcomes as reported by 45 studies. (**a**) Solid organ transplants caused by COVID-19 infection as reported by 34 studies. (**b**) Solid organ transplants post-COVID-19 (not caused by COVID-19) as reported by 9 studies. (**c**) Solid organ transplants in donors who had COVID-19 as reported by 2 studies. (NR: not reported).

The 45 studies included 1280 patients, of which 1279 underwent organ transplants at ages between 3 months and 77 years. Figure 3 shows a breakdown of the type of transplants different patients underwent within each category. While not all the studies reported the gender of the patients, 953 patients were reported as males and 317 as females. Death was reported in 253 patients, while 970 were reported as recovered or recovering at the latest follow-up time.

### 3.2. Clinical Data

The studies reported a total of 1279 organ transplants (lung, liver, kidney, or heart), of which 1107 patients required transplants due to organ damage caused by COVID-19 (Figure 3a). Moreover, 162 recipients required transplants post-COVID-19 infection due to non-COVID-19 causes (Figure 3b), and 10 patients received organs from donors who previously had COVID-19 (Figure 3c).

#### 3.2.1. Organ Transplant Due to Organ Damage Caused by COVID-19

The following sections summarize the reported data for patients who required lung, liver, or heart transplants due to organ damage caused by COVID-19 infection.

##### Lung Transplants Due to COVID-19

Table 1 outlines data from the 26 studies highlighting COVID-19-positive patients with COVID-19-induced lung damage necessitating a transplant. A total of 1090 patients from the included studies underwent lung transplants (LTs). The patient population was predominantly male, with 828 male patients and 250 females. The patients were aged from 18 to 77 years, and numerous different comorbidities were reported, including diabetes mellitus (DM), hypertension (HTN), hyperlipidemia (HLD), anemia, obesity, coronary artery disease (CAD), asthma, and others.

**Figure 4:**
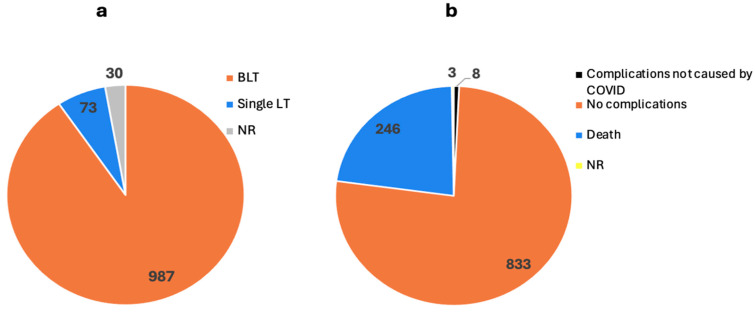
Types of lung transplants caused by COVID-19 infection and their outcomes as reported by 26 studies. The number on each section shows the number of patients. (**a**) Types of lung transplants. (**b**) The outcome of lung transplants. No complications related to COVID-19 were reported post-operatively. (BLT: bilateral lung transplant, LT: lung transplant, NR: not reported).

There were two different types of LTs (Figure 4a) reported in the studies: 987 bilateral lung transplants (BLTs) and 73 single LTs. This included living and deceased donors, and multiple lobar transplants involved donation from living family members. Most patients received standard immunosuppression of tacrolimus, mycophenolate mofetil, and steroids.

Several complications were documented in the studies, including cerebrovascular accidents (CVA), pneumothorax (PTX), pneumomediastinum, cardiac arrest, PE, DVT, gastrointestinal (GI) bleeding, sepsis, and many others. The mortality rate was 246/1090 (22.6%), and the rate of patients with explicitly reported recovery was 833/1090 (76.4%). These data are illustrated in Figure 4b. As will be elaborated upon in a later section, the data regarding recovery were limited. Therefore, this figure is based on our interpretation of the available information. The follow-up time ranged from 1 month to 1 year.

The severity of the infective episodes varied significantly, with some patients experiencing common, milder symptoms of fever, cough, headache, and fatigue, while others progressed to ARDS and irreversible pulmonary fibrosis requiring mechanical ventilation (MV), intubation, extracorporeal membrane oxygenation (ECMO), or intensive care unit (ICU) admission. The time from recovery following COVID-19 infection to transplant ranged from 41 to 381 days.

##### Liver Transplants Due to COVID-19

**Figure 5:**
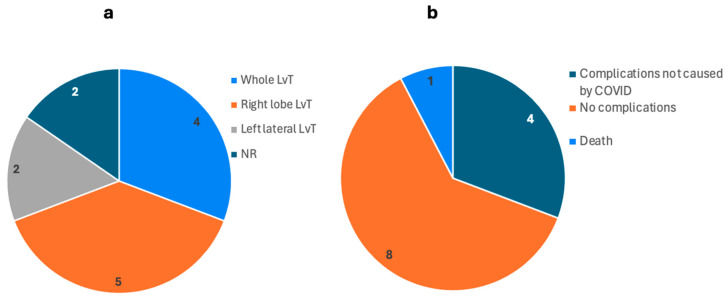
Types of liver transplants caused by COVID-19 infection and their outcomes as reported by 6 studies. The number on each section shows the number of patients. (**a**) Types of liver transplants. (**b**) The outcome of liver transplants. No complications related to COVID-19 were reported post-operatively. (LvT: liver transplant, NR: not reported).

Table 2 and Figure 5 detail the six studies examining liver transplantation (LvT) outcomes in patients who had previously contracted COVID-19. We included data from six studies encompassing case reports and retrospective case series, with a total of 38 patients.

The studies included in the analysis span different geographical locations including the USA, India, Turkey, and Israel. The patient demographic is predominantly male, with a gender distribution of 14.3% female and 85.7% male. The median age of the patients ranges from 35 to 64 years, reflecting a middle-aged population affected by severe liver complications following COVID-19.

Patients exhibited significant comorbidities such as DM, HTN, obesity, obstructive sleep apnea (OSA), HLD, and others. These comorbid conditions likely contributed to the severity of COVID-19 and subsequent liver damage.

The types of liver transplantation performed included both living donor liver transplant (ldLvT) and deceased donor liver transplant (ddLvT). Donor information varied, with some donors being living relatives.

All patients received standard immunosuppressive regimens post-transplantation, typically consisting of tacrolimus, mycophenolate mofetil, and steroids.

The studies documented several post-transplant complications including CVA, pneumomediastinum, cardiac arrest, PE, DVT, GI bleeding, and sepsis. Despite these complications, the overall outcomes were generally positive. Sambommatsue et al. [21] reported an 86% patient and graft survival rate at a median follow-up of 11 months, with only one patient dying 5 months after transplantation due to respiratory failure.

The severity of COVID-19 varied among patients, with many experiencing ARDS, requiring MV, and in some cases, ECMO. The median time interval from initial COVID-19 diagnosis to liver transplantation was reported to be 381 days, with a range of 210 to 820 days.

##### Heart Transplants Due to COVID-19

Table 3 outlines data on the two studies reporting heart transplants (HTs) in patients following infection with COVID-19. Of the five patients included in the studies, four (3M/1F) patients underwent HT.

The median age ranged from 38 to 46 years, and patient information was not available other than one case of chronic lymphopenia as a comorbidity.

Data were only available for one patient, who received standard initial immunosuppression of antilymphocyte serum, high-dose corticosteroids, and mycophenolate mofetil. The patient was later maintained on decreased doses of corticosteroids, mycophenolate mofetil, and cyclosporine.

**Figure 6:**
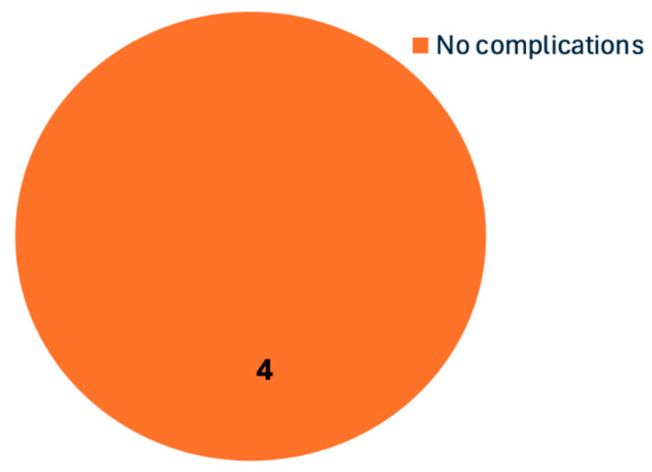
Heart transplants caused by COVID-19 infection and their outcomes in 4 patients as reported by 2 studies. No complications were reported.

Complications were not reported in the studies, and the recovery rate was 100% (Figure 6). Furthermore, the recovery time was not mentioned in the studies.

The reported cases were either asymptomatic or not severe, with symptoms including fever, vomiting, and SOB. Three patients were hospitalized for an average duration of 150 ± 113 days post-COVID-19 and were in New York Heart Association (NYHA) class IV on admission. The last patient had a case of post-COVID-19 fulminant myocarditis and was bridged on venoarterial ECMO (VA-ECMO) prior to undergoing HT 11 days after admission.

#### 3.2.2. Transplants Post-COVID-19 Infection (Not Caused by COVID-19)

Table 4 summarizes data on the nine studies involving patients infected with COVID-19 but with organ damage induced by non-COVID-19 causes. There was a total of 162 patients, with 104 being male and 58 being female: 37 underwent LvT, 124 underwent a kidney transplant (KT), and 1 patient had a dual kidney–pancreas transplant.

The median age ranged from 30 to 66 years, and patients had a wide variety of comorbidities, including DM, HTN, end-stage renal disease (ESRD), obesity, CAD, anemia, hypothyroidism, and several others.

**Figure 7:**
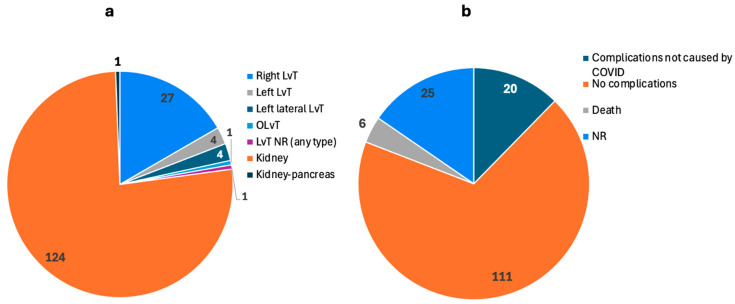
Types of solid organ transplants in patients post-COVID-19 infection (organ damage not caused by COVID-19) and their outcomes as reported by 9 studies. The number on each section shows the number of patients. (**a**) Types of transplants. (**b**) The outcome of transplants. No complications related to COVID-19 were reported post-operatively. (LvT: liver transplant, OLvT: orthotopic liver transplant, NR: not reported).

The transplants performed in the studies included LvT, KT, and dual kidney–pancreas transplant. Reported details on the types of LvTs can be seen in Figure 7a, which include 27 right lobe LvT, 4 left lobe LvT, 4 left lateral LvT, 1 orthotopic liver transplant (OLvT). Details on the other types of transplants were not available. Regarding donor information, there were both deceased and living donors with variable information.

The approach to immunosuppression varied between studies, with regimens being tailored to each patient. Some received standard initial immunosuppression of tacrolimus, high-dose corticosteroids, and mycophenolate mofetil. Many also received antibiotic prophylaxis, anticoagulation, and antiviral medication.

There were many reported complications within the studies, including acute and chronic rejection, early hepatic artery thrombosis, thrombocytopenia, leukopenia, mild pancreatitis, cytomegalovirus (CMV) infection, encephalopathy, urinary tract infection (UTI), and others. Although 20 of the 162 patients experienced significant complications, there were only six deaths (3.7%), and the recovery rate was 111/162 (68.5%) (Figure 7b). Information on recovery was not extensive within the studies, so this figure is based on our interpretation of the studies. Only a few studies reported follow-up duration, and the median range of follow-up was from 81 to 574 days post-op.

The severity of the reported COVID-19 infections ranged from mild to severe, and symptoms included fever, loss of appetite, cough, loss of taste, headache, fatigue, myalgia, and several other commonly known symptoms. Multiple patients required ICU admission and intubation, but the majority of cases were not as severe. Following recovery, patient time to transplant ranged from 2 weeks to 13 months.

#### 3.2.3. Transplants with Organs from Donors Who Had COVID-19

**Figure 8:**
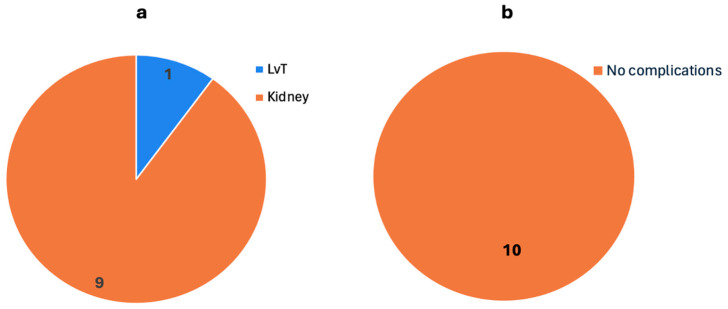
Types of transplants post-COVID-19 infection when the donor had COVID-19 as reported by 2 studies. The number on each section shows the number of patients. (**a**) Types of transplants. (**b**) The outcome of transplants. No complications were reported post-operatively. (LvT: liver transplant).

Table 5 outlines data on the two included studies where the donor had COVID-19. There were 10 patients in total in these studies with an equal gender distribution of 5 males and 5 females. Within this group, nine patients underwent KT, and one patient underwent LvT (Figure 8a).

The patient population was young, with a median age range from 25 to 45 years. Reported comorbidities include CKD, ESRD, Crohn’s disease, and primary sclerosing cholangitis (PSC), which are likely to be major contributing factors to why a transplant was required.

The LvT was a right lobe LvT. Of the 10 organ donors, the donor of the right lobe of the liver was the only living donor. The other eight donors were COVID-19-positive deceased donors, with seven being vaccinated and two being unvaccinated, who donated a total of nine kidneys.

As post-op immunosuppression, all patients received tacrolimus, mycophenolate, and steroids.

None of the patients had major post-op complications, graft loss, or donor-derived COVID-19 infection, and the recovery rate was 100% (Figure 8b).

Nine patients had 30-day follow-up following a median hospital stay of 4 days. Follow-up information on the last patient was not presented.

One live donor was suspected to have SARS-CoV-2 infection on pre-op day 3, although the nasal swab was negative, due to presenting with fever, tachycardia, and chest X-ray (CXR) changes supportive of COVID-19 infection. The negative nasal swab was assumed to be negative due to a short incubation time and was categorized as a false negative. The donor fully recovered 13 days post-op. Information on the severity of the infective episodes of the other eight donors was not presented in the studies.

## 4. Discussion

The tendency of SARS-CoV-2 to have detrimental effects on not only the respiratory system but also on multiple organ systems has been a well-documented phenomenon (for example, multisystem inflammatory syndrome in children (MIS-C)). This introduced several immunological unknowns regarding the mechanisms behind organ damage, potential correlations with disease severity, and long-term effects following infection with COVID-19. This organ damage may lead to solid organ transplantation, as reported by many studies.

Furthermore, a clinical consequence of acute COVID-19 infection that should not be overlooked is graft rejection in transplant recipients. These patients are especially susceptible to infections due to their medication-induced immunosuppressed state and are more likely to develop signs and symptoms of severe infection. Consequently, they are potentially at significant risk of graft rejection following infection with COVID-19. Several studies looked at the impact of COVID-19 infections on solid organ transplants that were conducted before infection, i.e., on the existing grafts at the time of infection. Alhumaid et al. investigated the outcomes of solid organ rejection following COVID-19 infection or vaccination, which revealed that 40 of the total 136 solid organ rejections were due to COVID-19 infection [61]. Most biopsies taken following rejection showed features consistent with acute cellular rejection of the corresponding organ. For example, 30 of the 40 cases were biopsied, and 23 of those biopsies were in cases of kidney rejection with the histopathology showing acute renal cellular rejection. There were 3 cases of mortality, with the remaining 37 cases surviving without any serious complications. The primary immune response responsible for the cases included in this study is the widely documented acute T-cell mediated organ injury [61]. Fenninger et al. reported that, compared to non-transplant patients, transplant patients with COVID-19 infection had a strong cellular response, seemingly to compensate for a mild humoral response. The immune profile of these patients was comparable to that of patients with severe COVID-19, and they had a significantly increased number of effector T cells and CD28- senescent T cells. They also had decreased naive T cells and, in terms of cytokines, had elevated CCL2 and reduced CCL4 [62].

**Figure 9:**
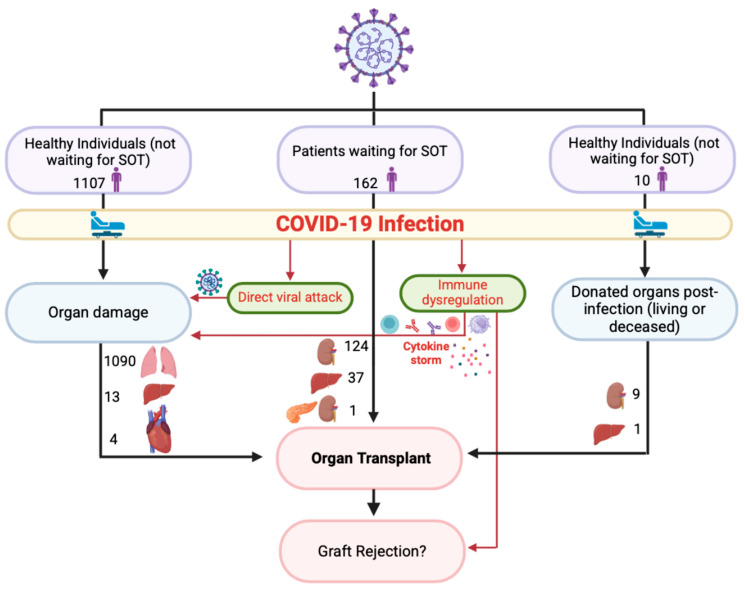
This study included 1279 patients who had solid organ transplants (SOTs) post-COVID-19 infection. Of the 1279 patients, 1107 had SOTs due to organ damage post-infection (1090 lung, 13 liver, 4 heart), 162 contracted COVID-19 while waiting for organ transplantation (124 kidney, 37 liver, 1 kidney and pancreas), and 10 donated organs post-COVID-19 infection (9 kidney and 1 liver).

The aim of this study is to analyze the existing data on organ transplants, such as success rates and clinical outcomes, where at least one organ, recipient, or donor is affected by COVID-19. This study mainly focuses on the operations that were conducted post-infection rather than before COVID-19 infection. This may highlight information in the existing literature regarding the various mechanisms behind COVID-19-induced organ damage and whether it has a significant impact on the outcomes of organ transplants to the point where perhaps a COVID-19-specific protocol should be developed. This approach allowed us to split the data into three categories, in the setting of organ transplants, based on whether the donor or the recipient was affected by COVID-19, and if organ damage was due to COVID-19 or other causes. This yielded a total of 45 studies, and we analyzed the outcomes of 1279 organ transplants where the lungs, kidneys, heart, or liver were involved based on the aforementioned scenarios. Of those transplants, 1107 involved recipients following COVID-19 infection and with organ damage due to COVID-19, 162 involved recipients following COVID-19 infection with organ damage due to non-COVID-19 causes, and 10 were cases where only the donor had COVID-19. In this way, we were able to assess each category separately and attempt to draw conclusions based on the outcomes. Figure 9 summarizes the aim and structure of this study.

### 4.1. Post-COVID-19 Recipient with Organ Damage Due to COVID-19

#### 4.1.1. Lung Transplants Caused by COVID-19 Infection

In the case where the recipient suffered from COVID-19 infection leading to severe lung damage necessitating a transplant, the main focus was investigating the mechanism behind the damage to the lungs and the effect on transplant outcomes.

This section contains the bulk of the studies within this category, which is no surprise as COVID-19 is primarily a respiratory pathogen. Specifically, 26 of the 45 included studies fell under this category, rendering a total of 1090 patients, of which 987 patients underwent a BLT and 73 underwent a single LT. The rest were not defined within the studies. Studies comparing transplant outcomes in COVID-19-positive to matched non-COVID-19-positive patients could not demonstrate a statistically significant difference between the two cohorts.

For example, Okumura et al. [31] conducted a retrospective cohort study looking at 1-year outcomes of LTs due to COVID-19-induced lung disease in a matched cohort of 268 patients. They found no difference between the cohorts in terms of primary graft failure, 30-day survival, 90-day survival, 1-year survival, COVID-19-related deaths, and overall patient mortality. This was further supported by a case series completed by Franco-Palacios et al. [29], which featured five cases of BLTs. The results supported that patients with COVID-19 ARDS have good short-term survival post-transplant, but the study could not provide data or comment on long-term survival [29]. Nonetheless, no data from the included studies supported the theory that COVID-19 patients had worse outcomes following LTs.

#### 4.1.2. Liver Transplants Caused by COVID-19 Infection

Liver injury post-COVID-19 infection was reported by several studies. The data presented in Table 2 provide a comprehensive overview of LvT outcomes in patients who sustained major liver damage in the post-COVID-19 setting. This section explores the potential role of COVID-19 in inducing liver damage as well as its influence on post-transplantation outcomes, particularly rejection.

The studies included a total of 38 patients with significant comorbidities such as DM, HTN, obesity, OSA, and HLD. These underlying conditions are known to exacerbate the severity of COVID-19 and likely contribute to the observed liver damage [23].

The outcomes post-LvT in these patients were generally positive. In Sambommatsue et al.’s [21] retrospective case series, six out of seven patients survived with good graft function at a median follow-up of 11 months, despite one patient succumbing to respiratory failure 5 months post-transplant. This highlights an 86% patient and graft survival rate, indicating that LvT can be a viable option for patients with COVID-19-induced liver damage.

Durazo et al. [23] presented a case of a patient with severe COVID-19 and subsequent liver failure who underwent OLvT and achieved normal allograft function 7 months post-transplant. This case demonstrates that full recovery post-transplant is possible with effective management, even in severe cases.

In the case report by Rela et al. [33], the patient underwent an auxiliary partial orthotopic liver transplant (APOLT) for severe cholangiopathy following COVID-19. The patient showed good graft function and recovery at the 6-month follow-up, with no rejection episodes reported. This suggests that while COVID-19 may induce severe liver damage, it does not necessarily predispose patients to higher rates of graft rejection. Complications post-transplantation included CVA, pneumomediastinum, cardiac arrest, PE, DVT, GI bleeding, and sepsis. Despite these complications, rejection episodes were notably rare. For instance, Durazo et al. [23] reported no episodes of acute cellular or antibody-mediated rejection in their case.

The data in the studies indicated that LvT is a viable and often necessary intervention for patients suffering from severe liver damage due to COVID-19. The outcomes post-transplant were generally favorable, with low incidences of graft rejection, though complications remain a significant concern.

The studies emphasized the importance of early detection and management of post-COVID-19 cholangiopathy, as well as the need for ongoing research on this topic to develop optimized management protocols, considering the evolving nature of COVID-19 variants and their potential impact on liver health.

#### 4.1.3. Heart Transplants Caused by COVID-19 Infection

This section discusses the data that pertain to COVID-19 patients requiring HTs due to the infection, and if there is a significant impact on post-op outcomes.

Post-transplant outcomes were favorable, and all four patients recovered well. Rossi-Neto et al. [18] conducted a prospective cohort study comparing outcomes between LC HT patients and non-COVID-19 HT patients. Given the sample size was small, with 4 LC patients and 41 non-COVID-19 patients, there was no significant difference in outcomes between the cohorts in terms of success rate and mortality. There was one patient death in the LC cohort prior to HT, rendering the amount of LC HTs to be three in the study, but no patient deaths after HT [18]. The other study was a case report by Gaudriot et al. [30], which highlighted a 38-year-old male patient who developed acute fulminant myocarditis post-COVID-19 and recovered well following HT. The outcomes of these studies are re-assuring, and they support HT as a treatment option for severe cardiac complications induced by COVID-19.

#### 4.1.4. Mechanism of Organ Damage by COVID-19

The etiology of COVID-19-induced organ damage remains unclear; however, several of the studies proposed possible mechanisms. These will be explored in the following section.

##### Mechanism of Lung Damage by COVID-19

In terms of lung injury, the following studies commented on possible etiologies based on test results pertaining to the patient population. First, a case study by Anderle et al. [16] emphasized a possible association between COVID-19-induced antibodies and interstitial lung disease (ILD) with respiratory failure, alongside direct COVID-19-related damage. The study featured a 20-year-old unvaccinated patient presenting with polyarthritis, exertional dyspnea, and fatigue for 3–4 weeks. The patient was found to have an elevated anti-melanoma differentiation-associated gene 5 (MDA5) autoantibody titer, evidence of myositis on MRI, and ground-glass opacities on lung CT scan. He was diagnosed with anti-MDA5 dermatomyositis (DeM). The study provided various possible explanations, but none established a definite causation. Nevertheless, the authors hypothesized that the COVID-19 infection may have triggered the production and viral induction of MDA5 antibodies, which, in turn, led to the development of DeM with rapidly progressive ILD. This is also supported by the corresponding chronological timelines available in the literature [63]. Moreover, a case series conducted by Uhl et al. [46] aimed to investigate the mechanisms underlying the exacerbation of lung fibrosis in idiopathic pulmonary fibrosis (IPF) patients following COVID-19 infection, focusing on the role of TGFB1. The study concluded that the role of both BMP and TGFB1 signaling in the pathogenesis of IPF and post-COVID-19 fibrosis (PCF) may have implications for the development of future treatments.

The role of ACE-2 receptors in mediating organ changes is a frequently discussed mechanism in the literature. These receptors serve as a target for cell entry into lung epithelial and alveolar cells, primarily type II, after which viral replication leads to cell death. As previously mentioned, this is not the main contributor to organ damage, and it is rather the robust immune response and widely documented “cytokine storm” that leads to inflammatory changes and tissue damage [64]. This leads to the typical ARDS manifestations and fibrosis that, if severe, can lead to lung transplants being required.

##### Mechanism of Liver Damage by COVID-19

The etiology behind liver injury seemed to be discussed with less uncertainty in the included studies. Some of the primary mechanisms through which COVID-19 causes liver damage include direct viral cytopathic effects, immune-mediated damage, and ischemic injury. The presence of the ACE-2 receptor, which SARS-CoV-2 uses to enter the cells, in cholangiocytes is one potential pathway for direct viral damage. Moreover, severe inflammatory responses and cytokine storms associated with COVID-19 can result in significant liver injury [21]. This multifactorial pathophysiology is evident in the patients included in these studies, many of whom developed secondary sclerosing cholangitis in critically ill patients (SSC-CIP) [33].

##### Mechanism of Heart Damage by COVID-19

With myocarditis and other cardiac complications being commonly reported in COVID-19 cases, the heart is now known to be a commonly targeted organ by the virus. Unfortunately, given the small sample size and study aims not aligning with this learning objective, there was no insight into the mechanism behind COVID-19-induced heart complications.

However, the past few years yielded studies showing that ACE-2 receptors and the immune response are, much like the other organs, key mediators of COVID-19-induced heart damage. Myocardial cells express ACE-2 receptors, which are a target for viruses. This leads to direct cell death and cytotoxicity, as well as the immune response following infection inducing an inflammatory state. Infection with COVID-19 also leads to a hypercoagulable state, and the aforementioned mechanisms are major contributors to the reported cases of MI, myocarditis, and thrombotic events [65].

### 4.2. Transplants Post-COVID-19 Infection (Not Caused by COVID-19)

This discussion covers the data regarding patients with COVID-19 infection, but with organ damage not induced by the virus. The goal behind including this category is to evaluate if COVID-19 infection has an effect on organ transplant outcomes where organ damage is due to non-COVID-19 causes and if a correlation can be made with the impact of the virus on the immune system.

In terms of post-op outcomes, the studies showed positive results that did not differ from the general population. In a retrospective observational study, Akbulut et al. [50] highlighted 35 patients who underwent LvT following COVID-19 infection and compared them to 439 non-COVID-19 patients in terms of post-op morality. There was no statistical significance between the two cohorts (see the section below), and the authors concluded that exposure to COVID-19 does not affect mortality following organ transplants. Kute et al. [54,55] also conducted two retrospective cohort studies where they analyzed outcomes in post-KT patients following COVID-19 infection, with one of the studies having 100% patient and graft survival in a cohort of 75 patients. Their results showed that after comprehensive screening and careful pre-transplant evaluation, KTs post-COVID-19 can safely be performed with standard immunosuppression protocols without increased risk of complications. The other included studies also had favorable outcomes, showing that COVID-19 has no significant impact on post-op outcomes in KTs and LvTs.

As mentioned in the results, there were several complications within the studies, including encephalopathy, UTI, CMV infection, and others. There were also multiple cases of graft rejection in the studies. For example, Akbulut et al. [50] reported four patients from a COVID-19 cohort with graft rejection (three acute, one chronic) at a median of 25 days post-LvT. Following the transplant, five (14.3%) deaths were observed, with the causes of mortality being cardiac problems in four patients and mesenteric ischemia in one patient. Notably, none of the deaths were associated with complications due to COVID-19 infection. Kute et al. [54] also reported an acute rejection rate of 13.1%, a graft loss rejection rate of 2.6%, and one patient death 6 months post-op due to fungal pyelonephritis following KT. However, these data were not statistically significant when compared to a non-COVID-19 cohort, further supporting organ transplant outcomes as independent of COVID-19 infection exposure.

### 4.3. Transplants with Organs from Donors Who Had COVID-19

This section highlights the setting in which the donor had COVID-19, with a close look at the effect on transplant outcomes and the immune system.

Post-transplant outcomes were overwhelmingly positive in the included studies. Sanchez-Vivaldi et al. [59] reported a nine-patient case series where all patients were free from dialysis and had satisfactory allograft function on the 30-day follow-up. Nguyen et al.’s [60] case report presented a 24-year-old male patient who underwent right lobe LvT from a living donor. The patient was reported to have excellent post-op outcomes. The outcomes of these studies show that patients can receive organs from COVID-19-positive donors without increased risk of graft rejection or other complications.

As mentioned in the results, both Sanchez-Vivaldi et al. [59] and Nguyen et al. [60] reported no major post-transplant complications, graft loss, or donor-derived infections in the follow-up period. Even though a sample size of 10 patients is small, it is based on a young population with equal gender distribution. If more studies with even larger patient populations can be performed and yield similar results, then this can significantly support the theory that it is safe to perform organ transplants from COVID-19-positive donors.

### 4.4. Impact of Previous COVID-19 Infection on Transplant Outcomes

Understanding the effects of COVID-19, both short- and long-term, on the immune system is a scientific challenge, but there has been a surge in studies in recent years aimed at investigating those effects and how they affect transplant outcomes. We included studies with data on patients with a history of infection with COVID-19 and analyzed post-transplantation complications, mortality, and the causes behind it, which are outlined in this section.

#### 4.4.1. Impact on Lung Transplant Outcomes—Recipient COVID-19+, Organ Damage by COVID-19

There were several reported complications and patient deaths following LT. For example, the case series conducted by Franco-Palacios et al. [29] reported mortality due to septic shock 5 months post-BLT. Similarly, Reis et al. [32] reported two patient deaths due to fungal sepsis almost 2 months post-LT (whole). Those patients also had to receive high-dose steroids for management of grade 2 acute cellular rejection prior to the septic episodes. Other studies also reported mortality due to fungal sepsis and recurrent infections. Almost all the studies that reported immunosuppression protocol involved the use of standard triple immunosuppression (for example, tacrolimus, mycophenolate mofetil, and glucocorticoids). Though the role of immunosuppressants can certainly not be ignored, sepsis and recurrent infections as common causes of mortality in multiple similar studies should probe more research questions pertaining to the effect of COVID-19 on the immune system.

#### 4.4.2. Impact on Liver Transplant Outcomes—Recipient COVID-19+, Organ Damage by COVID-19

Prior COVID-19 infection did not significantly affect the outcomes of LvTs regarding rejection. However, the long-term impact on overall health and the potential for ongoing complications remains a concern. The persistence of elevated liver enzymes and bilirubin levels post-recovery from COVID-19 was a common observation, necessitating close monitoring and timely intervention [40]. The cases of pediatric patients described by Cooper et al. [49] further illustrate the potential for severe liver injury even in mild COVID-19 cases, leading to the need for LvT. The proposed mechanisms of liver injury include autoimmune responses and dysregulated immune responses post-COVID-19 infection, contributing to conditions such as secondary hemophagocytic lymphohistiocytosis (HLH) [49].

#### 4.4.3. Impact on Heart Transplant Outcomes—Recipient COVID-19+, Organ Damage by COVID-19

Patients with COVID-19 infection who required HT had favorable outcomes following organ transplant, and one study noted a pattern of injury to the heart. When compared to non-COVID-19 cohorts, Rossi-Neto et al. [18] noted that patients in the COVID-19 group seemed to have characteristics consistent with low cardiac output rather than congestion. It was also noted that the short-term mortality rate of the patients with LC after undergoing HT was low. In addition, they emphasized the important role of vaccinations in mitigating post-infection complications and mortality. Gaudriot et al. [30] also recommended taking a closer look at VA-ECMO as a bridge to HT in COVID-19-associated myocarditis. Even though there were only two studies included regarding this topic, the data are re-assuring, and the authors are in favor of HT as a treatment option for COVID-19-induced heart injury when necessary. However, more research should be performed on this topic in terms of exploring the reasons behind COVID-19 leading to severe cardiac complications.

#### 4.4.4. Impact on Transplant Outcomes—Recipient COVID-19+, Organ Damage by Non-COVID-19 Causes

In cases where organ damage was not due to COVID-19 in COVID-19-positive patients, the data seem to be in favor of, with careful assessment tailored to each case, transplants not being negatively impacted by a positive history of COVID-19 infection in the recipient. Akbulut et al. [50] concluded that exposure to, and infection with, COVID-19 before LvT has no effect on patient and graft survival post-op. When it comes to vaccinated patients, Antal et al. [51] concluded that KT can be safely performed earlier than is recommended in post-COVID-19 asymptomatic patients with lower mortality rates. Similarly, Kute et al. [54] mentioned that, with standard immunosuppression protocols, ABOiKTx in post-COVID-19 candidates can be safely performed as well. In terms of recovery time, the shortest and longest reported recovery time was 30 days and 91 days, respectively. Overall, the included studies yielded results that show COVID-19 has no significant impact on post-transplant outcomes in terms of graft survival, mortality, and complications. Certainly, this is not a definite conclusion, and more studies should be conducted and analyzed, but the data so far seem to show favorable outcomes for different types of organ transplants.

#### 4.4.5. Impact on Transplant Outcomes—Donor COVID-19+

Although only two studies were included, studies where the authors covered organ transplants where only the donor had COVID-19 had promising outcomes. Both studies demonstrated significantly favorable results. Not only was the recovery rate 100% (10/10), but the mortality rate was 0%. The shortest reported recovery period was 30 days. Sanchez-Vivaldi et al. [59] concluded that it is possible to use organs from SARS-CoV-2-positive donors and have satisfactory outcomes. The outcome of the case report performed by Nguyen et al. [60] was also excellent and supports this conclusion. It is also re-assuring that no changes or adjustments in standard immunosuppression regimens were required in any of the reported cases, which shows that it is possible to approach organ transplants from both COVID-19-positive and non-COVID-19 donors in the same manner. We acknowledge that only a couple of studies met our inclusion criteria within this category, but the current data warrant optimism for future outcomes, and more studies should be conducted.

### 4.5. Effect of COVID-19 Infection on the Outcome of Organ Transplants

COVID-19 infection has a significant impact and influence on the immune profiles of patients during the convalescence period. Despite this dysregulation, no significant effect was reported on the outcome of solid organ transplantation post-COVID-19 infection. This could be linked to the length of the period between recovery and organ transplantation. Studies have noted differences between different stages of the convalescent period. Silva-Junior et al. conducted a longitudinal study that compared the immune profiles of healthy donors and convalescent patients using serum and venous blood samples. Typically, the convalescence period showed high levels of IL-15, the neutrophil-to-lymphocyte ratio, and red blood cell distribution width (RDW). In the initial stage, a high absolute monocyte and basophil count, platelet count, myeloid dendritic cell count, and anti-Spike IgG level were observed. From 30 days to 60 days, there was increased production of patrolling monocytes, B1 lymphocytes, G-CSF, IL-2, and a high IFN-γ/IL4 ratio. After 60 days (late convalescence), the pattern shifted to a reparative and proliferative dynamic, which may be a response to the inflammatory damage that occurred during the acute phase of COVID-19 infection. There was an increased number of total and activated cytotoxic lymphocytes, plasmacytoid dendritic cells, vascular endothelial growth factor (VEGF), IL-9, and chemokine CXCL-10 [66]. It was also noted that the convalescence period, in general, involved an increased level of inflammatory and patrolling monocytes. The activated inflammatory and patrolling subpopulations were seen to increase in proportion, even as the total monocyte levels were seen to regulate. This increase was seen up to 2 months after viral clearance but then decreased and stabilized in around 5 months. Inflammation regulation and tissue repair may be the likely reason for this observation. Additionally, the cytotoxic element seen during the convalescent period was seen to be regulated by T-lymphocytes. NK and NKT cells were seen to be persistently decreased during the acute phase of infection and convalescence. This decrease was also associated with disease severity and cytotoxicity compromise. Myeloid and plasmacytoid dendritic cell levels showed no difference, implying that their role is dependent on stimuli and that they have a rapid role in viral clearance [66]. It was also noted that the resolution of lung pathology correlated with the recovery of IL-10+ B-cells. These cells may have a role in inflammation suppression and improved long-term outcomes [67].

### 4.6. Study Limitations

This study has some limitations. Firstly, several studies were ambiguous when it came to clearly reporting if patients were recovering, fully recovered, or recovering with complications. This led to us using our own interpretation when categorizing patients, which may have skewed some calculations or resulted in different conclusions. Moreover, data on whether complications were due to COVID-19 or non-COVID-19 causes were quite minimal, which made it difficult to come to a final conclusion on the impact that COVID-19 has on clinical outcomes following organ transplants. In addition, information on vaccination status was limited in the studies, but this is likely due to many studies being initiated prior to vaccine distribution or to patients being ineligible at the time the vaccine was available. In terms of study exclusion, much like the approach of many other studies, we employed methods of quality control and excluded all conference abstracts to avoid redundant data that were published in journals as well. Moreover, the majority of the included studies were either case studies or case reports, with the 8 included cohort studies being the least of the total 45 studies. This has its pros and cons, as we ended up with a relatively small patient population, but we also obtained more detailed conclusions and recommendations tailored for the patients highlighted in case reports/series. Another limitation was the lack of information about the donors in some studies, especially whether the donors were living or deceased, and many of the studies that reported such information did not separate the outcome based on the type of donor. This did not allow us to determine the effect of this factor on the outcome of transplantation. Finally, there was a disproportionate number of studies relevant to each category. For example, in the category where patients had COVID-19 infection and organ damage due to COVID-19, there were 26 studies about LTs, but only 6 studies for LvTs and 2 for HTs. Certainly, this is most likely due to COVID-19 being primarily a respiratory pathogen, but it does not change the fact that some categories may have had too few studies and small patient populations, which makes some conclusions limited in terms of data analyzed.

## 5. Conclusions and Recommendations

COVID-19 has been identified as a multifaceted disease affecting multiple organs, including the lungs, liver, kidneys, and heart. Based on the included studies, we obtained a better idea regarding the mechanisms behind COVID-19-induced organ damage and its impact on post-transplantation outcomes.

Lungs: Regarding LTs, studies that compared COVID-19 to non-COVID-19 cohorts yielded results supporting no statistical difference in post-op clinical outcomes. For this reason, Reis et al. [32], Okumura et al. [31], and Lang et al. [42] believed in the surgical feasibility of LTs and recommended lung transplantation as a treatment option with excellent prognostic value, especially in the setting of post-COVID-19 ARDS. In another case study, Sajid et al. [20] also concluded that lung transplantation is a life-saving treatment for patients with COVID-19-associated irreversible respiratory failure and pulmonary fibrosis while emphasizing the need for more research and guidelines regarding lung transplantation in the context of COVID-19, specifically focusing on the timing of transplantation in relation to the infection, negative PCR testing, and negative Vero cell cultures in cases of persistent positive COVID-19 PCR testing. Similarly, Mortazavi et al. [43] stated that additional research is required to explore and investigate the underlying mechanisms, the treatment efficacy of steroids and immunosuppression, and the development of guidelines for evaluating the eligibility of patients to undergo bilateral orthotopic lung transplantation (BOLT). As mentioned in the Discussion, the case study performed by Anderle et al. [16] mentioned the formation of antibodies following COVID-19 infection, leading to the negative outcomes seen in the lungs. Uhl et al. [46] also recommended investigating the role of BMP and TGFB1 in IPF and PCF to better guide the development of treatments.

Liver: The data from the included studies also provided valuable insights into the management and outcomes of liver transplantation in patients with COVID-19-induced liver damage. The findings suggest that with appropriate management and follow-up, liver transplantation can be a successful intervention for this patient population, despite the significant challenges posed by their underlying health conditions and the severity of their COVID-19 infection. The retrospective case series by Sambommatsue et al. [21] had excellent outcomes at the 11-month follow-up, which led the authors to conclude that liver transplantation is a viable treatment option following COVID-19-induced liver damage. This was also supported by the outcomes in the study performed by Akbulut et al. [50], where they found no significant statistical difference in mortality between COVID-19 and non-COVID-19 liver transplant cohorts and concluded that COVID-19 had no effect on patient and graft survival after undergoing a liver transplant. In addition, Durazo et al. [23] highlight that even in severe cases, full recovery is achievable with proper management post-transplant after undergoing OLvT.

Kidneys: In terms of KTs, Antal et al. [51] believed it is safe to perform KT earlier than recommended in asymptomatic patients following infection with COVID-19. Kute et al. [54] also believed in the safety of ABOiKT without needing to deviate from standard immunosuppression protocols.

Heart: The two included studies by Gaudriot et al. [30] and Rossi-Neto et al. [18] had outcomes that support HT as a viable treatment option post-COVID-19 without needing to adjust current treatment protocols. In addition, Gaudriot et al. [30] recommend investigating VA-ECMO and perhaps using it more frequently as a bridge to HT in the case of COVID-19-associated myocarditis.

Donors with COVID-19: When organ donors are those with COVID-19 infection, Sanchez-Vivaldi et al. [59] and Nguyen et al. [60] suggested that organ transplants in this setting can be performed and have positive outcomes. Moreover, changes to the existing approach to immunosuppression were not required in any case, which is re-assuring in terms of being able to treat organ transplants where COVID-19 is implicated in a usual manner and still expect positive outcomes.

Overall, the current literature warrants optimism regarding COVID-19 and organ transplants, but more studies should be conducted to help expand on this information, especially paying attention to other organs, with a focus on the detailed reporting of clinical outcomes and analyzing the impact of COVID-19 on these procedures.

## Figures and Tables

**Table 1:** Lung transplants caused by COVID-19 infection.

Authorde	Study Type/ Country	N (Total) Gender (F%/M%)	Age Mean ± SE/ Median (IQR) (Years)	Type of Organ Transplant	Information About the Donor	Outcome
Anderle et al. [16]	Case report Austria	1 M	20	BLT	NA	In remission for 12 months
Rohr et al. [17]	Case report USA	1 M	31	ddBLT	Deceased donor	Recovering well
Rossi et al. [19]	Case report Italy	1 M	18	BLT	NA	Progressing well during the first month post-Tx
Sajid et al. [20]	Case study USA	1 M	43	BLT	NA	Discharged on day 89 Recovering well
Bermudez et al. [22]	Retrospective cohort study USA	305 65 F 240 M (21.3%, 78.7%)	51 (42–57)	279 (91%): BLT 9: dual organ Tx (7 lung–kidney + 2 heart–lung)	Median (IQR) donor age: 33 (23–43) years	Post-Tx survival: 97% at 1 month; 94.3% at 6 months; 87.1% at 12 months
Florissi et al. [24]	Retrospective cohort study USA	353 79 F 274 M (22%, 78%)	51 (40–57)	325 BLT 28 single LT	Median (IQR) age: 33 (24–43) years	Alive at 30-day follow-up (out of available data for 281): 273 Alive at 90-day follow-up (out of available data for 214): 202
Gogia et al. [25]	Case report India	1 M	34	BLT	Donor was blood group- and size-matched to patient Deceased donor	Discharged on POD 15 Recovering and was undergoing rigorous therapy at POD 250
Schwarz et al. [26]	Retrospective cohort analysis Austria	40 9 F 31 M (22.5%/77.5%)	SSC Group (n = 15): 57 (42–61) Non-SSC Group (n = 25): 54 (44.5–56)	SSC Group: 7 ddBLT 7 size reduction 1 lobar LT Non-SSC Group: 19 ddBLT 2 size reduction 4 lobar LT	Median age: 50 years Deceased donors	One-year survival rate: 90% (non-SSC group); 47% (SSC); eight deaths (five due to SSC)
Shah et al. [27]	Retrospective case series India	23 3 F 20 M (13%/87%)	42 (34–58)	BLT	NA	Died due to the following: Sepsis: eight Neurologic CVA: one CMV infection: one
Shimizu et al. [28]	Case report Japan	1 F	57	ldLT	Right lower lobe from son Left lobe from husband	Recovered and was transferred to a rehabilitation hospital on POD 131
Franco-Palacios et al. [29]	Case series USA	5 2F 3M (40%/60%)	37 (33–54)	BLT	Deceased brain death donors	Died due to septic shock: one No clinically significant acute allograft rejection: four
Okumura et al. [31]	Retrospective cohort study USA	COVID-19 cohort: 268 (26%/74%) Non-COVID-19 cohort: 268 (24%/76%)	COVID-19 cohort: 53.0 (44.0–59.0) Non-COVID-19 cohort: 55.0 (44.0–61.0)	LT Single LT: COVID-19 cohort: 9 Non-COVID-19 cohort: 26 BLT: COVID-19 cohort: 239 Non-COVID-19 cohort: 242	Donors’ median age: in the COVID-19 cohort: 33 years Non-COVID-19 cohort: 34 years Deceased donors	[COVID-19 vs. non-COVID-19 cohort, *p*-value] No difference between both cohorts regarding primary graft failure [0 (0%) vs. 1 (0.4%), 0.99], 30-day [7 (2.6%) vs. 9 (3.4%), 0.61], 90-day [9 (3.4%) vs. 15 (5.6%), 0.21], 1-year [22 (8.2%) vs. 26 (9.7%), 0.55], COVID-19-related [2 (0.7%) vs. 0 (0%), 0.50], and overall patient deaths [22 (8.2%) vs. 34 (13%), 0.09]
Reis et al. [32]	Cohort study Brazil	3 1 F 2 M	P1: 46 (M) P2: 34 (F) P3: 31 (M)	BLT	Donors: M (19, 34, 21 years, respectively) Deceased donors	P1 and P2 died due to fungal sepsis POD on 47 and 52, respectively P3 was discharged at POD 30
Roda et al. [34]	Case report Italy	1 M	63	BLT	NA	Died in 06/2021, after another septic episode
Hall et al. [35]	Case study USA	1 F	52	BLT	NA	Quickly improved and discharged on POD 14
Haslbauer et al. [36]	Case report Austria	1 M	62	BLT	NA	Intensive rehabilitative program Discharged two months post-Tx
Herrmann et al. [37]	Case report USA	1 F	28	BLT	NA	Successful Tx Discharged on POD 21
Javaid et al. [38]	Retrospective case series USA	2 F 4 M (33%/66%)	55	BLT	36 years	
		P1 M	69		P1: 39F Deceased donor	
		P2 M	63		P2: 40F Deceased donor	
		P3 F	47		P3: 49M Deceased donor	
		P4 M	58		P4: 28F Deceased donor	
		P5 M	32		P5: 22M Deceased donor	
		P6 F	62		P6: 37F Deceased donor	
Kehara et al. [39]	Retrospective case series USA	20 3 F 17 M (15%/85%)	Mean: 58 ± 12 62 (31–77)	Double LT (1 was REDO): 7 RLT (1 was w/CABG) *: 8 LLT (1 was w/CABG) *: 5	Age: 38± 14 years 38 (14–61) years 3 donors w/diabetes 1 smoker	Death due to COVID-19-related myocarditis on POD 195: 1/20 COVID-19 recurrence after LT on POD 149, 257, and 326, alive: 3/20 Improvement in native lung: 5/13 single LT
Koch et al. [41]	Case report Germany	1 M	31	BLT	Age: 28 years M with ICH Deceased donor	Successful lung BLT Discharged 96 days after surgery
Lang et al. [42]	Retrospective analysis Austria	19 (16%/84%)	56 (34–64)	BLT	NA	Died post-Tx (fully functional grafts): five P1, 2, and 3: Died PODs 65, 66, and 154 due to liver failure P 4: Died POD 111 due to recurrent infections P 5: Died POD 147 after being discharged (spontaneous ICH) Early post-operative outcome was worse in patients who developed ARDS due to COVID-19
Mortazavi et al. [43]	Case series USA	20 (40%/60%)	39 (24–66)	BOLT	NA	Three died on POD 13, 19, and 305 Overall, 17/20 were alive up to POD 495 despite COVID-19-related complications prior to OT
Ohsumi et al. [44]	Case report Japan	1 F	57	ldLLT	Right lower lobe: Patient’s son Left lower lobe: Patient’s husband	Four months post-Tx: no complications noted
Umemura et al. [45]	Case report Japan	1 F	50	ldLLT	NA	POD 117, patient was CMV-positive and was transferred to rehabilitation on POD 131
Uhl et al. [46]	Case series USA	12 NA	NA	LT	NA	NA
Magnusson et al. [48]	Case report Sweden	1 M	55	BLT	NA	Successful BLT No signs of infection or Tx rejection Discharged on POD 34

ARDS: acute respiratory distress syndrome, BLT: bilateral lung transplant, BOLT: bilateral orthotopic lung transplantation, CABG: Coronary Artery Bypass Graft, CMV: cytomegalovirus, COVID-19: Coronavirus Disease 2019, CVA: Cerebral Vascular Accident, ddBLT: Deceased Donor Bilateral Lung Transplant, ICH: Intracerebral Hemorrhage, IQR: Interquartile Range, ldLLT: Living Donor Lobar Lung Transplantation, ldLT: Living Donor Lung Transplant, LLT: Left Lung Transplant, LT: lung transplant, NA: Not Applicable/Mentioned, POD: post-operative day, RLT: Right Lung Transplant, SSC: secondary sclerosing cholangitis, Tx: transplant/transplantation. * Some patients had intraoperative CABG because of obstructive lesions.

**Table 2:** Liver transplants caused by COVID-19 infection.

Author	Study Type/ Country	N (Total) Gender (F%/M%)	Age Mean ± SE/ Median (IQR) (Years)	Type of Organ Transplant	Information About the Donor	Outcome
Sambommatsue et al. [21]	Retrospective case series USA	7 1 F 6 M (14.3%/85.7%)	61	Whole LvT: 3 Right lobe LvT: 4	Four living donors Three deceased donors	One died due to respiratory failure 5mo post-op Six recovering well (patient with longest f/u 18 mos) Patient and graft survival rate at a median f/u of 11 mos is 86%
Durazo et al. [23]	Case report USA	1 M	47	OLvT (whole)	Deceased donor	Discharged on POD 55 Normal allograft function 7 mos after Tx
Rela et al. [33]	Case study India	1 M	50	Right lobe APOLvT	Living donor: Daughter	Discharged on POD 9 Recovering well with good graft function at 6 mos
Kiyak et al. [40]	Case report Turkey	1 M	35	ldLvT	Living donor	All LFTs were normal within 3 mos of Tx
Lee et al. [47]	Case report USA	1 M	64	LvT	Deceased donor	Discharged on POD 4. No complications at 8 mos f/u
Cooper et al. [49]	Retrospective case series Israel	2 M				
		P1	3 mo	Live left lateral LvT	Father	Recovered well
		P2	5 mo	Live left lateral LvT	Mother	Recovered well

APOLvT: auxiliary partial orthotopic liver transplantation, COVID-19: Coronavirus Disease 2019, F/U: follow-up, IQR: Interquartile Range, ldLvT: living donor liver transplant, LFT: Liver Function Test, LvT: liver transplant, MOS: months, OLvT: orthotopic liver transplant, POD: post-operative day, Tx: transplant/transplantation.

**Table 3:** Heart transplants caused by COVID-19 infection.

Author	Study Type/ Country	N (Total) Gender (F%/M%)	Age Mean ± SE/ Median (IQR) (Years)	Type of Organ Transplant	Information About the Donor	Outcome
Rossi-Neto et al. [18]	Prospective cohort study Brazil	45		HT		
		COVID-19 group: 4 (25%/75%)	COVID-19 group: 46.1 ± 15.7	HT	NA	COVID-19 group: 0/3 deaths in patients after HT
		Non-COVID-19 group: 41 (31.7%/68.3%)	Non-COVID-19 group: 38.2 ± 9.1	HT	NA	Non-COVID-19 group: 2/15 deaths in patients after HT
Gaudriot et al. [30]	Case report France	1 M	38	HT	NA	Recovered well

COVID-19: Coronavirus Disease 2019, HT: heart transplant, IQR: Interquartile Range, NA: Not Applicable/Mentioned.

**Table 4:** Solid organ transplants post-COVID-19 (organ damage not caused by COVID-19).

Author	Study Type/ Country	N (Total) Gender (F%/M%)	Age Mean ± SE/ Median (IQR) (Years)	Type of Organ Transplant	Information About the Donor	Outcome
Akbulut et al. [50]	Observational retrospective study Case series Turkey	35 (34.3%/65.7%)	50 (95% CI 43–54)	LvT Liver graft type: Right: 27 Left: 4 Left lateral: 4	Living donor: 3 Deceased donor: 2	Post-Tx rejection: four at a median 25 days after LvT Dead: five within median 23 days after LvT Post-op f/u: median 574 days (95% CI 454–637) Exposure to, and infection with, COVID-19 before liver transplant does not affect the patients post-transplant or graft survival
Antal et al. [51]	Case report Romania	1 M	47	KT	Cadaver donor	Discharged on POD 28
Singh et al. [52]	Case report USA	1 F	66	Simultaneous PT and KT	NA	Discharged on POD 5 Asymptomatic at 2 months f/u
Sherwood et al. [53]	Case report Canada	1 M	30	ldKT	Living	Discharged on POD 7 Symptoms fully resolved by POD 30
Kute et al. [54]	Retrospective cohort study India	38 32 F 6 M	38.5 (31.25–47.5)	KT	All donors were living females	One death at 6 months post-op due to fungal pyelonephritis Acute rejection: 13.1% Graft loss: 2.6%
Kute et al. [55]	Retrospective cohort study India	75 8 F 67 M	39.4 ± 12, 40 (7–62)	KT	52 F/23M Age: 47 ± 10, 47 (29–72)	Patient and graft survival: 100% F/U duration: 91 ± 47, 81 (56–117) days
Jacob et al. [56]	Case report Australia	1 F	39	LvT	Deceased donor	Discharged on POD 14 Recovered by POD 30
Okubo et al. [57]	Case report USA	1 M	65	OLvT	NA	Discharged with home health services Last f/u on POD 30. COVID-19 PCR -ve.
Juric et al. [58]	Retrospective case series Croatia	9 5 M 4 F	40.8 (18–71)	KT	Eight deceased donors	NA

CI: Confidence Interval, COVID-19: Coronavirus Disease 2019, F/U: follow-up, IQR: Interquartile Range, KT: kidney transplant, ldKT: Living Donor Kidney Transplant, LvT: liver transplant, OLvT: orthotopic liver transplant, PCR: Polymerase Chain Reaction, POD: post-operative day, PT: pancreas transplant, Tx: transplant/transplantation.

**Table 5:** Solid organ transplants when the donor had COVID-19.

Author	Study Type/ Country	N (Total) Gender (F%/M%)	Age Mean ± SE/ Median (IQR) (Years)	Type of Organ Transplant	Information About the Donor	Outcome
Sanchez-Vivaldi et al. [59]	Retrospective case series USA	9 5 F 4 M (55.6%/44.4%)	45.0 (32.0–54.0)	KT	13 SARS-CoV-2 deceased donors 28.6% F 71.4% M	No complications Median hospital stay of 4 days All recipients had satisfactory allograft function (median creatinine of 1.51 mg/dL) at 30-day follow-up
Nguyen et al. [60]	Case report USA	1 M	24	Right Lobe LvT	Live donor	Excellent post-op outcome

COVID-19: Coronavirus Disease 2019, IQR: Interquartile Range, KT: kidney transplant, LvT: liver transplant, SARS-CoV-2: Severe Acute Respiratory Syndrome Coronavirus 2.

## Data Availability

The data that supports the findings of this study are available in the Supplementary Materials of this article.

## Supplementary Materials

The following supporting information can be downloaded at: https://www.mdpi.com/article/10.3390/biomedicines13020428/s1, Table S1: Demographic and clinical data for the reported patients who underwent organ transplant post-COVID-19 or received the organ from a donor who had COVID-19; File S1: Database search strategy.
