# Supplementary material for: Solid Organ Transplants Caused by COVID-19 Infection and the Outcome of Transplantation Post-COVID-19: A Systematic Review
Source: Biomedicines. 2025 Feb 10;13(2):428. doi: 10.3390/biomedicines13020428 (PMC11852956; doi:10.3390/biomedicines13020428)
Supplement: Supplementary file 1 [file biomedicines-13-00428-s001.zip › biomedicines-3387853-Supplementray File S1.pdf]

File S1: Information sources and database search strategy.

PubMed | October 2023

"Long COVID"[tiab:~3] OR "Post Covid"[tiab:~3] OR "Chronic Covid"[tiab:~3] OR "Covid Sequelae"[tiab:~3] OR "Persistent COVID"[ti:~3] OR "Long Coronavirus"[tiab:~3] OR "Post Coronavirus"[tiab:~3] OR "Chronic Coronavirus"[tiab:~3] OR "Coronavirus Sequelae"[tiab:~3] OR "Persistent Coronavirus"[ti:~3] OR "Persistent COVID"[ti:~3] OR "PASC" OR "convalescent COVID"[tiab:~3] OR "convalescent Coronavirus"[tiab:~3] OR "Post-Acute COVID-19 Syndrome"[Mesh]

Medline (Ovid - 1946 – Current) | October 2023

long COVID/ OR ((Long ADJ3 Covid) OR (Chronic ADJ3 Covid) OR (Post ADJ3 Covid) OR (COVID ADJ3 Sequelae) OR (Long ADJ3 Coronavirus) OR (Chronic ADJ3 Coronavirus) OR (Coronavirus ADJ3 Sequelae) OR (Persistent ADJ3 Coronavirus) OR (Persistent ADJ3 COVID) OR (convalescent ADJ3 COVID) OR (convalescent ADJ3 Coronavirus) OR (Post ADJ3 Coronavirus) OR PASC).ti,ab.

Embase (Ovid 1974 – 2023) | October 2023

long COVID/ OR ((Long ADJ3 Covid) OR (Chronic ADJ3 Covid) OR (Post ADJ3 Covid) OR (COVID ADJ3 Sequelae) OR (Long ADJ3 Coronavirus) OR (Chronic ADJ3 Coronavirus) OR (Coronavirus ADJ3 Sequelae) OR (Persistent ADJ3 Coronavirus) OR (Persistent ADJ3 COVID) OR (convalescent ADJ3 COVID) OR (convalescent ADJ3 Coronavirus) OR (Post ADJ3 Coronavirus) OR PASC).ti,ab.

Scopus | October 2023

TITLE-ABS((Long W/3 Covid) OR (Chronic W/3 Covid) OR (Post W/3 Covid) OR (COVID W/3 Sequelae) OR (Long W/3 Coronavirus) OR (Chronic W/3 Coronavirus) OR (Coronavirus W/3 Sequelae) OR (Persistent W/3 Coronavirus) OR (Persistent W/3 COVID) OR (convalescent W/3 COVID) OR (convalescent W/3 Coronavirus) OR (Post W/3 Coronavirus) OR PASC) OR INDEXTERMS ((long W/3 covid) OR "Post-Acute COVID-19 Syndrome")

Web of Science | October 2023

(TI=((Long NEAR/3 Covid) OR (Chronic NEAR/3 Covid) OR (Post NEAR/3 Covid) OR (COVID NEAR/3 Sequelae) OR (Long NEAR/3 Coronavirus) OR (Chronic NEAR/3 Coronavirus) OR (Coronavirus NEAR/3 Sequelae) OR (Persistent NEAR/3 Coronavirus) OR (Persistent NEAR/3 COVID) OR (convalescent NEAR/3 COVID) OR (convalescent NEAR/3 Coronavirus) OR (Post NEAR/3 Coronavirus) OR PASC) OR AB=((Long NEAR/3 Covid) OR (Chronic NEAR/3 Covid) OR (Post NEAR/3 Covid) OR (COVID NEAR/3 Sequelae) OR (Long NEAR/3 Coronavirus) OR (Chronic NEAR/3 Coronavirus) OR (Coronavirus NEAR/3 Sequelae) OR (Persistent NEAR/3 Coronavirus) OR (Persistent NEAR/3 COVID) OR (convalescent

NEAR/3 COVID) OR (convalescent NEAR/3 Coronavirus) OR (Post NEAR/3 Coronavirus) OR PASC) OR KP=(Long NEAR/3 COVID))

Science Direct | October 2023

"Long COVID" OR "Post Covid" OR "Chronic Covid" OR "Post-Acute COVID-19 Syndrome" OR "Post-Acute Sequelae of SARS-CoV-2 Infection" OR "post COVID-19 syndrome" OR "chronic COVID syndrome" OR "Post COVID-19 Syndrome" OR
"Post-COVID"

Cochrane Library | October 2023

("Long COVID" OR "Post Covid" OR "Chronic Covid" OR "Post-Acute COVID-19 Syndrome" OR "Post-Acute Sequelae of SARS-CoV-2 Infection" OR "post COVID-19 syndrome" OR "chronic COVID syndrome" OR "Post COVID-19 Syndrome" OR "Post-COVID"):ti,ab,kw AND MeSH descriptor: [Post-Acute COVID-19 Syndrome] explode all trees
