# Supplementary material for: Solid Organ Transplants Caused by COVID-19 Infection and the Outcome of Transplantation Post-COVID-19: A Systematic Review
Source: Biomedicines. 2025 Feb 10;13(2):428. doi: 10.3390/biomedicines13020428 (PMC11852956; doi:10.3390/biomedicines13020428)
Supplement: Supplementary file 1 [file biomedicines-13-00428-s001.zip › biomedicines-3387853-Supplementray Table S1.pdf]

Supplementary Table S1: Demographic and clinical data for the reported patients who underwent organ transplant post-COVID-19 or received the organ from a donor who had COVID-19.

Study	Study Type<br><br>Country	N (total)<br>Gender<br>(F%/M%)	Age<br>Mean ±SE/<br>Median<br>(IQR)<br>(years)	Comorbidities<br>(recipient)	COVID<br>Date/<br>duration<br>severity	Type of organ<br>transplant	How long<br>after COVID	Organ<br>donor	Management	Complication<br>and any<br>clinical<br>characteristics	Outcome	Score<br><br>QA type
Studies reporting transplants caused by COVID-19 infection												
Anderle et al.[16]	Case report<br><br>Austria	1 M	20	None	NA<br><br>Mild<br><br>Elevated body temperature<br>Cough<br>General weakness	BLT<br><br>Excellent primary organ function with removal of ECMO at the end of the operation	2.5 months after suspected COVID-19 infection	NA	Standard triple immunosuppression:<br>Tacrolimus<br>mycophenolate mofetil<br>glucocorticoids<br><br>Physiotherapy<br><br>Transferred to a rehabilitation center and given further outpatient care	ANA and anti-MDA5 Ab negative at 1 and 5 months after transplant<br><br>Anti-Ro-60 Ab marginally elevated (12 U/mL) at 1 month after transplant and negative at 5 months after transplant	In remission for 12 months	6/8<br><br>Murad et al.[15]
Rohr et al.[17]	Case report<br><br>USA	1 M	31	Grade 3 Obesity	NA<br><br>First positive COVID-19: 3 months prior to Tx evaluation (tested negative)	ddBLT	140 days after the initial COVID-19 diagnosis	Deceased donor	NA	None	Recovering well<br>Continuing rehabilitation similar to a non-COVID-19 transplant recipient	3/8<br><br>Murad et al.[15]

Study	Study Type<br><br>Country	N (total)<br>Gender (F%/M%)	Age<br>Mean ±SE/ Median (IQR) (years)	Comorbidities (recipient)	COVID Date/ duration severity	Type of organ transplant	How long after COVID	Organ donor	Management	Complication and any clinical characteristics	Outcome	Score<br><br>QA type
Studies reporting transplants caused by COVID-19 infection												
Rossi-Neto et al.[18]	Prospective cohort study<br><br>Brazil	45			NA	HT	NA	NA	NA	NA	NA	6/9<br><br>NOS[14]
		COVID group:<br><br>4 (25%/75%)	COVID group: 46.1±15.7	COVID group:<br><br>HoS<br>Obesity	NA	HT	NA	NA	NA	NA	COVID group:<br><br>0/3 deaths in patients after HT	
		Other group:<br><br>41<br><br>(31.7%/68.3%)	Other group: 38.2±9.1	Other group:<br><br>Arterial HTN<br>DM<br>HoS<br>Obesity	NA	HT	NA	NA	NA	NA	Other group:<br><br>2/15 deaths in patients after HT	
Rossi et al.[19]	Case report<br><br>Italy	1 M	18	NA	NA	LT	NA	NA	Tx occurred after 71 days of MV and 55 days of VV-ECMO<br><br>Patient was placed in immunosuppression regimens and isolated for 15 days	NA	Progressing well throughout the physiotherapy sessions and achieving the expected	3/8<br><br>Murad et al.[15]

Study	Study Type<br><br>Country	N (total)<br>Gender<br>(F%/M%)	Age<br>Mean ±SE/<br>Median<br>(IQR)<br>(years)	Comorbidities<br>(recipient)	COVID<br>Date/<br>duration<br>severity	Type of organ<br>transplant	How long<br>after COVID	Organ<br>donor	Management	Complication<br>and any<br>clinical<br>characteristic<br>s	Outcome	Score<br><br>QA type
Studies reporting transplants caused by COVID-19 infection												
											goals<br>during the<br>first<br>month<br>post<br>transplant	
Sajid et al.[20]	Case study<br><br>USA	1 M	43	AS<br>HTN<br>GERD<br>HLD	NA<br><br>Admission 8<br>days after<br>testing<br>positive due<br>to:<br>Hypoxia<br>Worsening<br>SOB	LT	Day 71 of<br>hospital stay	NA	Immunosuppressi<br>on:<br>Pulse steroids<br>post-surgery<br>tacrolimus<br>mycophenolate<br>mofetil<br>Mycophenolate<br>mofetil (switched<br>to azathioprine<br>due to diarrhea)<br><br>Other<br>treatments:<br>vasopressin<br>epinephrine<br>norepinephrine<br>meropenem<br>cefazoline<br>micafungin for<br>pneumonia and<br>AKI	Developed<br>septic shock<br>post-Tx<br>because of<br>donor-<br>associated<br>pneumonia<br>and AKI	Discharge<br>d on day<br>89<br><br>Recoverin<br>g well	5/8<br><br>Murad et<br>al.[15]
Sambomma<br>tsue et al.[21]	Retrospecti<br>ve case<br>series	7<br><br>1 F<br>6 M	61	DM<br>HTN	03/2020 (F)<br>04/2020<br>(M)	LvT	Median time<br>interval from<br>initial COVID-<br>19 diagnosis	4: ldLvT<br>3: ddLvT<br>(donation	All patients<br>received a<br>standard<br>immunosuppressi	CVA<br>PTX<br>Pneumomedi<br>astinum	1 patient<br>died 5<br>months<br>after LvT	5/8<br><br>Murad et<br>al.[15]

Study	Study Type<br><br>Country	N (total)<br>Gender<br>(F%/M%)	Age<br>Mean ±SE/<br>Median<br>(IQR)<br>(years)	Comorbidities<br>(recipient)	COVID<br>Date/<br>duration<br>severity	Type of organ transplant	How long after COVID	Organ donor	Management	Complication and any clinical characteristic<br>s	Outcome	Score<br><br>QA type
Studies reporting transplants caused by COVID-19 infection												
	USA	(14.3%/85<br>.7%)			07/2020<br>(M)<br>08/2020<br>(M)<br>09/2020<br>(M)<br>09/2020<br>(M)<br>05/2021<br>(M)<br><br>7: ARDS<br>6: MV<br>1: ECMO		to LvT: 381<br>days<br>(210–820<br>days)	after brain<br>death)	ve regimen<br>consisting of:<br>tacrolimus<br>mycophenolate<br>mofetil<br>steroids	Cardiac arrest<br>PE<br>DVT<br>GI Bleed<br>Sepsis	due to<br>respirator<br>y failure<br><br>6 patients<br>are alive<br>and doing<br>well<br><br>Patient<br>and graft<br>survival<br>rate at a<br>median<br>follow up<br>of 11<br>months is<br>86%	
Bermudez<br>et al.[22]	Retrospecti<br>ve cohort<br>study<br><br>USA	305<br><br>65 F<br>240 M<br>(21.3%,<br>78.7%)	51 (42-57)	DM<br>HoS<br>Dialysis pre-Tx<br>Previous<br>cardiac surgery<br>Lung surgery<br>between listing<br>and transplant	NA<br><br>61.6%:<br>ARDS<br>38.4%:<br>pulmonary<br>fibrosis<br>237: ICU<br>158: MV<br>129:<br>Tracheosto<br>my<br>191: ECMO	279 (91%):<br>BLT<br><br>9: dual organ<br>Tx (7 lung–<br>kidney + 2<br>heart–lung)	NA	Median<br>(IQR) donor<br>age: 33 (23-<br>43) years	Post-Tx:<br>230 (75.4%):<br>Ventilation for<br>>48 hours<br><br>79 (25.9%):<br>Postoperative<br>ECMO<br><br>36 (12.3%): New<br>dialysis	Acute<br>transplant<br>rejection<br>(n=25)<br><br>Bronchial<br>dehiscence<br>(n=7)<br><br>CVA (n=7)<br><br>Median<br>hospital stay:<br>28 days	34/305<br>patients<br>(11.1%)<br>died<br>during<br>follow-up<br><br>Post-Tx<br>survival:<br>97% at 1<br>month<br><br>94.3% at<br>6 months	9/9<br><br>NOS[14]

Study	Study Type<br><br>Country	N (total)<br>Gender (F%/M%)	Age<br>Mean ±SE/ Median (IQR) (years)	Comorbidities (recipient)	COVID Date/ duration severity	Type of organ transplant	How long after COVID	Organ donor	Management	Complication and any clinical characteristics	Outcome	Score<br><br>QA type
Studies reporting transplants caused by COVID-19 infection												
											87.1% at 12 months<br><br>1-year survival post-Tx did not differ between patients with ARDS and pulmonary fibrosis	
Durazo et al.[23]	Case report<br><br>USA	1 M	47	Obesity class 3 (BMI 51)<br>OSA<br>HTN<br>HLD	Severe COVID-19 with:<br>ARDS<br>AKI<br><br>MV (29 days)<br>Continuous VV hemofiltration<br>Liver failure with SSC (day 81)	OLvT	Tx on day 108 from initial presentation	Deceased donor<br><br>OLvT whole hepatic allograft	Induction immunosuppression:<br>Basiliximab (simulect)<br>Solumedrol taper<br>everolimus (Zortress)<br><br>Maintenance therapy:<br>Tacrolimus<br>everolimus<br>rehabilitation from day 46	No complications related to Tx surgery<br><br>required regular hemodialysis and was undergoing evaluation for a renal transplant	Normal allograft function 7 months after Tx.<br><br>No acute cellular or antibody-mediated rejection of the transplanted liver<br><br>discharge	4/8<br><br>Murad et al.[15]

Study	Study Type<br><br>Country	N (total)<br>Gender<br>(F%/M%)	Age<br>Mean ±SE/<br>Median<br>(IQR)<br>(years)	Comorbidities<br>(recipient)	COVID<br>Date/<br>duration<br>severity	Type of organ transplant	How long after COVID	Organ donor	Management	Complication and any clinical characteristics	Outcome	Score<br><br>QA type
Studies reporting transplants caused by COVID-19 infection												
											d on POD 55	
Florissi et al.[24]	Retrospective cohort study<br><br>USA	353<br><br>79 F<br>274 M<br>(22%, 78%)	51 (40-57)	Previous malignancy<br>DM<br>HoS<br>Prior cardiac surgery<br>Pre-Tx dialysis	NA<br><br>41: 10% functional status (moribund) pre-Tx<br>189: 20% functional status (very sick) pre-Tx122: Other functional status pre-Tx	325 double LT<br>28 single LT	NA	Median (IQR) age was 33 (24-43) years	80: ECMO at 72 hours after Tx	CVA<br>Dialysis post-Tx<br><br>Median (IQR) length of stay following transplant is 26 (18-45) days	273: Alive at 30-day follow-up (out of available data for 281)<br><br>202: alive at 90-day follow-up (out of available data for 214)	8/9<br><br>NOS[14]
Gogia et al.[25]	Case Report<br><br>India	1 M	34	Previously healthy	Tested positive on 9/272021<br><br>Severe hypoxemia<br>O2 sat: 92% on 100% FiO2<br>MV<br>Femoro-jugular VV-ECMO	Double LT	Tx was done 41 days after recovery	Donor was blood group- and size-matched to the patient<br><br>Donor died in an RTA (brain-dead)	NA	NA	Discharged on POD 15<br><br>Recovering and was undergoing rigorous therapy at POD 250 at the time the paper was	3/8<br><br>Murad et al.[15]

Study	Study Type<br><br>Country	N (total)<br>Gender<br>(F%/M%)	Age<br>Mean ±SE/<br>Median<br>(IQR)<br>(years)	Comorbidities<br>(recipient)	COVID<br>Date/<br>duration<br>severity	Type of organ<br>transplant	How long<br>after COVID	Organ<br>donor	Management	Complication<br>and any<br>clinical<br>characteristic<br>s	Outcome	Score<br><br>QA type
Studies reporting transplants caused by COVID-19 infection												
					Remained<br>positive for<br>COVID-19<br>for 23 days						written	
Schwarz et<br>al.[26]	Retrospecti<br>ve cohort<br>analysis<br><br>Austria	40<br><br>9 F<br>31 M<br>(22.5%/77<br>.5%)	SSC Group<br>(n=15): 57<br>(42-61)<br><br>non-SSC<br>Group<br>(n=25): 54<br>(44.5-56)	NA	NA<br><br>100%:<br>ARDS<br>requiring LT	Double LT<br><br>Whole lungs<br>(n=26)<br>Size reduction<br>(n=9)<br><br>Lobar (n=5)<br><br>Whole lungs<br>(n=26):<br><br>SSC group<br>(n=7)<br><br>non-SSC<br>group (n=19)<br><br>Statistically<br>significant<br>difference<br>between the<br>two groups	Median time<br>between<br>initial COVID-<br>19 diagnosis<br>and being<br>listed for Tx:<br>49.5 days<br>(SSC group)<br>57.5 days<br>(non-SSC<br>group)<br><br>Waiting time<br>on the list:<br>Approx. 7<br>days for both<br>groups	Median age<br>of donors:<br>50<br><br>Causes of<br>death:<br>CI<br>ICH<br>TBI<br>Others<br><br>9 donors<br>had a Hx of<br>aspiration	vvECMO via<br>cannulation<br><br>LMWH/UFH were<br>used for<br>anticoagulation<br>during ECMO<br>bridging<br><br>Low tidal volume<br>ventilation	HTX<br><br>ECMO circuit<br>clotting<br><br>Vein<br>thrombosis<br><br>Median<br>postoperative<br>MV: 15 days<br><br>Median ICU<br>stay: 34.5-36<br>days	1-year<br>survival<br>rate:<br>90% (non-<br>SSC<br>group)<br><br>47% (SSC)<br><br>8 deaths<br>(5<br>due to<br>SSC)	8/9<br><br>NOS[14]
Shah et al.[27]	Retrospecti<br>ve case<br>series	23<br><br>3 F<br>20 M	42 (34-58)	HTN<br>DM<br>CAD	NA<br><br>Severe<br>post-COVID-	BLT	NA	NA	100%: BLT<br>following optimal<br>medical<br>management for	At 72 hours<br>post-Tx:<br>PGD (Grade 2)<br>PGD (Grade 3)	Died due<br>to:<br>Sepsis: 8<br>Neurologi	3/8<br><br>Murad et<br>al.[15]

Study	Study Type<br><br>Country	N (total)<br>Gender<br>(F%/M%)	Age<br>Mean ±SE/<br>Median<br>(IQR)<br>(years)	Comorbidities<br>(recipient)	COVID<br>Date/<br>duration<br>severity	Type of organ<br>transplant	How long<br>after COVID	Organ<br>donor	Management	Complication<br>and any<br>clinical<br>characteristic<br>s	Outcome	Score<br><br>QA type
Studies reporting transplants caused by COVID-19 infection												
	India	(13%/87%<br>)			19 fibrotic<br>progressive<br>ESLD<br>requiring<br>BLT				all patients<br><br>Postoperatively<br>immunosuppressi<br>on regimen:<br>Steroids<br>Tacrolimus<br>Mycophenolate<br>mofetil	Mean<br>hospital stay<br>post-Tx:<br>62.74±32.42<br>days	c CVA: 1<br>CMV<br>infection:<br>1	
Shimizu et<br>al.[28]	Case Report<br><br>Japan	1 F	57	NA	NA<br><br>Severe<br>post-COVID-<br>19 lung<br>injury	ldLT	3 months<br>after disease<br>onset	Right lower<br>lobe from<br>son<br><br>Left lobe<br>from<br>husband	Cefozopran<br>Levofloxacin<br>Micafungin<br>(perioperatively<br>for a week)<br>Heparin was not<br>administered due<br>to concerns about<br>postoperative<br>hemorrhage<br><br>Patient was<br>transfused with:<br>Packed red cells<br>Fresh frozen<br>plasma Platelet<br>concentrates	Anesthesia<br>and operation<br>times: 812<br>and 657 min,<br>respectively<br><br>Estimated<br>blood loss:<br>12,370 mL<br><br>Urine output:<br>1410 mL	Discharge<br>d from<br>the ICU<br>on POD<br>26<br><br>Weaned<br>from MV<br>approx. 2<br>months<br>post-Tx<br><br>Recovere<br>d and<br>was<br>transferre<br>d to a<br>rehabilitat<br>ion<br>hospital<br>on POD<br>131	5/8<br><br>Murad et<br>al.[15]

Study	Study Type<br><br>Country	N (total)<br>Gender<br>(F%/M%)	Age<br>Mean ±SE/<br>Median<br>(IQR)<br>(years)	Comorbidities<br>(recipient)	COVID<br>Date/<br>duration<br>severity	Type of organ<br>transplant	How long<br>after COVID	Organ<br>donor	Management	Complication<br>and any<br>clinical<br>characteristic<br>s	Outcome	Score<br><br>QA type
Studies reporting transplants caused by COVID-19 infection												
Franco-<br>Palacios et<br>al.[29]	Case Series<br><br>USA	5<br><br>2F<br>3M<br>(40%/60%<br>)	37 (33 - 54)	Asthma<br>Postpartum<br>UC	Between<br>8/2020 and<br>9/2021<br><br>End-stage<br>irreversible<br>COVID-19<br>pneumonia<br>and ARDS<br>leading to<br>single organ<br>(lung)<br>failure	BLT	Median (IQR)<br>time from<br>COVID-19 to<br>Tx: 54 days<br>(55.5 - 88)	Donated<br>organs<br>were from<br>deceased<br>brain death<br>donors<br><br>Negative<br>SARS-CoV-2	Systemic<br>corticosteroids<br>(dexamethasone<br>or<br>methylprednisolo<br>ne)<br><br>V remdesivir<br><br>Standard ICU care<br>for ARDS<br><br>VV-ECMO as a<br>bridge to lung Tx<br><br>Postoperative<br>ECMO<br>decannulation	Complications<br>of one<br>patient:<br><br>PGD<br><br>Hemorrhagic<br>pancreatitis<br><br>DVT<br>Right main<br>bronchus<br>anastomosis<br>dehiscence<br><br>Pseudoaneur<br>ysm of left<br>colic artery<br><br>Empyema<br><br>Septic shock<br>secondary to<br>GN<br>bacteremia<br>and<br>candidemia	1: Died<br>due to<br>septic<br>shock<br>secondary<br>to GN<br>bacteremi<br>a and<br>candidem<br>ia 5<br>months<br>after BLT<br><br>4: No<br>clinically<br>significant<br>acute<br>allograft<br>rejection<br>in<br>surveillan<br>ce<br>bronchosc<br>opies	6/8<br><br>Murad et<br>al.[15]
Gaudriot et<br>al.[30]	Case report<br><br>France	1 M	38	Chronic<br>lymphopenia<br><br>Non-smoker	5 weeks<br>before<br>admission<br>on<br>4/27/2020:	HT	Day 11 after<br>admission	NA	Standard<br>immunosuppressi<br>on:<br><br>Antilymphocyte	NA	Fully<br>recovered<br><br>Discharge<br>d for	3/8<br><br>Murad et<br>al.[15]

Study	Study Type<br><br>Country	N (total)<br>Gender (F%/M%)	Age<br>Mean ±SE/ Median (IQR) (years)	Comorbidities (recipient)	COVID Date/ duration severity	Type of organ transplant	How long after COVID	Organ donor	Management	Complication and any clinical characteristics	Outcome	Score<br><br>QA type
Studies reporting transplants caused by COVID-19 infection												
					Fever<br>SOB<br>Vomiting<br><br>4 weeks before admission: All resolved without treatment				serum<br>High-dose corticosteroids<br>Mycophenolate mofetil<br><br>Maintenance immunosuppression:<br><br>Decreasing dose of corticosteroids<br>Mycophenolate mofetil<br>Cyclosporine		cardiovascular rehabilitation without persistent respiratory or cardiac symptoms	
Okumura et al.[31]	Retrospective cohort study<br><br>USA	COVID cohort: 268 (26%/74%)<br><br>Non-COVID cohort: 268 (24%/76%)	COVID cohort: 53.0 (44.0-59.0)<br><br>Non-COVID cohort: 55.0 (44.0-61.0)	No difference between cohorts regarding:<br>DM<br>Serum creatinine<br>HCV serostatus<br>-On hemodialysis at the time of Tx<br>HoS	NA<br><br>COVID cohort:<br>ARDS<br>Pulmonary fibrosis	LT<br><br>Single LT:<br>COVID cohort: 9<br>Non-COVID cohort: 26<br><br>Double LT:<br>COVID cohort: 239<br>Non-COVID cohort: 242	NA	Donors' median age:<br><br>in the COVID cohort: 33<br><br>Non-COVID Cohort: 34<br><br>ddLT: Donor died due to:<br>Anoxia<br>CVA<br>Head trauma<br>CNS tumor	NA	No difference between both cohorts regarding complications such as:<br>PGD<br>Airway dehiscence<br>CVA<br>Organ rejection<br>Need for dialysis and pacemaker	No difference between both cohorts regarding graft survival and 30-day, 90-day, 1-year, COVID-19 related and overall patients	9/9<br><br>NOS[14]

Study	Study Type<br><br>Country	N (total)<br>Gender (F%/M%)	Age<br>Mean ±SE/ Median (IQR) (years)	Comorbidities (recipient)	COVID Date/ duration severity	Type of organ transplant	How long after COVID	Organ donor	Management	Complication and any clinical characteristics	Outcome	Score<br><br>QA type
Studies reporting transplants caused by COVID-19 infection												
								Other causes			deaths	
Reis et al.[32]	Cohort study<br><br>Brazil	3<br><br>1 F<br>2 M	P1: 46 (M)<br><br>P2: 34 (F)<br><br>P3: 31 (M)	None	NA<br><br>ICU admission Intubation<br><br>Later acquired irreversible pulmonary fibrosis	LT (whole)	NA	Donors: M (19, 34, 21 yo, respectively)<br><br>Donors of P1 and P3 had a HoS<br><br>Cause of death for donors:<br>P1 and P2: TBI<br>P3: CVA<br><br>All had normal bronchoscopy and pO2 /FiO2 ratios of up to 300<br><br>Brain death donor Tx	P1 and P2 received high-dose steroids for management of acute cellular rejection (grade 2)<br><br>Laparoscopic cholecystectomy for acute cholecystitis (P1)	Acute cellular rejection (grade 2) (P1 and P2)<br><br>Critical illness neuropathy (P1, P2 and P3)<br>Acute cholecystitis (P1)<br><br>Complicated pleural effusion (P3)	P1 and P2 died due to fungal sepsis POD on 47 and 52, respectively.<br><br>P3 was discharged at POD 30	8/9<br><br>NOS[14]
Rela et al.[33]	Case study<br><br>India	1 M	50	NA	NA	Right lobe APOLvT	6 weeks from recovery to presentation with	Living donor<br><br>Daughter of the patient	Standard thromboprophylaxis<br>LMWH	NA	Discharged on POD 9	6/8<br><br>Murad et al.[15]

Study	Study Type<br><br>Country	N (total)<br>Gender<br>(F%/M%)	Age<br>Mean ±SE/<br>Median<br>(IQR)<br>(years)	Comorbidities<br>(recipient)	COVID<br>Date/<br>duration<br>severity	Type of organ<br>transplant	How long<br>after COVID	Organ<br>donor	Management	Complication<br>and any<br>clinical<br>characteristic<br>s	Outcome	Score<br><br>QA type
Studies reporting transplants caused by COVID-19 infection												
					Severe<br>COVID-19<br>pneumonia<br><br>MV		progressively<br>worsening<br>cholestasis	Underwent<br>robotic<br>right lobe<br>donor<br>hepatectom<br>y			Good<br>graft<br>function<br>and<br>recoverin<br>g function<br>in native<br>liver<br>remnant<br>at 6<br>months<br>follow up	
Roda et<br>al.[34]	Case report<br><br>Italy	1 M	63	Hx of arterial<br>HTN<br><br>No Hx of liver<br>disease	11/2020<br><br>Presented<br>with:<br>Cough<br>Fever<br>Dyspnea<br>Radiological<br>evidence of<br>lung<br>consolidatio<br>ns<br>Positive RT-<br>qPCR test<br>for SARS-<br>CoV-2<br><br>Developed<br>ARDS and<br>required	BLT	COVID-19<br>infection in<br>November<br>2020<br><br>BLT in January<br>2021	NA	High bilirubin<br>levels and<br>abnormal LFTs:<br>Cytosorb filter<br>Plasma exchange-<br>cycles (partial<br>improvement)<br><br>HLH:<br>Dexamethasone<br>First-line therapy<br>(methylprednisol<br>one plus IgG)<br>Second-line<br>therapy<br>(Anakinra)<br><br>BAL (KPC XDR):<br>Meropenem/vab<br>orbactam	ICU stay was<br>complicated<br>by:<br>AKI III<br>Critical illness<br>polyneuropat<br>hy<br>Several<br>episodes of<br>bacterial<br>superinfectio<br>ns<br>PLS<br>Severe<br>haemolysis,<br>requiring<br>multiple<br>transfusions<br>with red cells	Died in<br>June<br>2021,<br>after<br>another<br>septic<br>episode	6/8<br><br>Murad et<br>al.[15]

Study	Study Type<br><br>Country	N (total)<br>Gender (F%/M%)	Age<br>Mean ±SE/ Median (IQR) (years)	Comorbidities (recipient)	COVID Date/ duration severity	Type of organ transplant	How long after COVID	Organ donor	Management	Complication and any clinical characteristics	Outcome	Score<br><br>QA type
Studies reporting transplants caused by COVID-19 infection												
					intubation and a VV-ECMO				Fever with CMV-DNA: Ganciclovir (complicated by neutropenia)<br><br>Patient had three sepsis episodes (last one was fatal)	Clinical course was complicated by:<br>HLH<br>Covid-19 SSC<br>Several episodes of septic shock by MDR opportunistic bacterial infections		
Hall et al.[35]	Case Study<br><br>USA	1 F	52	Healthy and exercised daily	Severe COVID-19 pneumonia progressing to ARDS in 7/2020 requiring BIPAP and CPAP<br><br>Developed progressive pulmonary fibrosis and required ECMO and received an LTt	BLT	Acute COVID infection in 7/2020<br><br>LT in 10/2020	NA	NA	NA	Extubated on POD 2<br><br>Quickly improved and discharged on POD 14	6/8<br><br>Murad et al.[15]

Study	Study Type<br><br>Country	N (total)<br>Gender<br>(F%/M%)	Age<br>Mean ±SE/<br>Median<br>(IQR)<br>(years)	Comorbidities<br>(recipient)	COVID<br>Date/<br>duration<br>severity	Type of organ transplant	How long after COVID	Organ donor	Management	Complication and any clinical characteristic<br>s	Outcome	Score<br><br>QA type
Studies reporting transplants caused by COVID-19 infection												
Haslbauer et al.[36]	Case Report<br><br>Austria	1 M	62	HTN<br>HLD<br>DM (type II)	12/2020<br><br><br>Presented with acute dyspnea in February 2021 (COVID PCR negative)<br><br>COVID-19 associated with lung fibrosis due to mild COVID infection 3 months earlier	BLT	3 months	NA	NA	NA	Intensive rehabilitat ive program<br><br>Discharge d two months post-Tx	6/8<br><br>Murad et al.[15]
Herrmann et al.[37]	Case Report<br><br>USA	1 F	28	Neuromyelitis optica on immunosuppre ssion	Admitted on 4/26/2020<br><br>Hospitalizati on for 66 days due to acute hypoxemic respiratory failure due	BLT	Patient was admitted on 4/26/2020 and subsequently hospitalized for 66 days until Tx	NA	Intensive physical therapy<br><br>Occupational therapy<br><br>Speech language pathology	NA	Successful transplant ation<br><br>Underwe nt inpatient rehabilitat ion	6/8<br><br>Murad et al.[15]

Study	Study Type<br><br>Country	N (total)<br>Gender<br>(F%/M%)	Age<br>Mean ±SE/<br>Median<br>(IQR)<br>(years)	Comorbidities<br>(recipient)	COVID<br>Date/<br>duration<br>severity	Type of organ<br>transplant	How long<br>after COVID	Organ<br>donor	Management	Complication<br>and any<br>clinical<br>characteristic<br>s	Outcome	Score<br><br>QA type
Studies reporting transplants caused by COVID-19 infection												
					to COVID-<br>19<br><br>MV<br>ECMO						Discharge<br>d on POD<br>21	
Javaid et<br>al.[38]	Retrospecti<br>ve case<br>series<br><br>USA	2 F<br>4 M<br><br>(33%/66%<br>)	55		Data<br>collected<br>from<br>August<br>2020<br>onward<br><br>Duration =<br>days until<br>first neg<br>PCR	BLT	NA	36<br><br>No chest<br>trauma to<br>any<br><br>No smokers<br><br>PaiO2/Fio2:<br>482<br><br>CMV<br>positive for<br>all donors<br>except for<br>P3<br><br>COVID<br>negative<br><br>CXR normal<br>in P1, P5	Tacrolimus-based<br>immunosuppressi<br>on,<br>mycophenolate<br><br>Prednisone<br><br>Post transplant<br>prophylaxis for<br>CMV and PCP<br><br>COVID<br>vaccination:<br>2 patients got 3<br>doses<br>3 patients got 2<br>doses 1 patient<br>got 1 dose	5 patients<br>survived with<br>excellent<br>functional<br>scores<br><br>One patient<br>died from<br>post-<br>transplant<br>infection		5/8<br><br>Murad et<br>al.[15]

Study	Study Type<br><br>Country	N (total)<br>Gender<br>(F%/M%)	Age<br>Mean ±SE/<br>Median<br>(IQR)<br>(years)	Comorbidities<br>(recipient)	COVID<br>Date/<br>duration<br>severity	Type of organ transplant	How long after COVID	Organ donor	Management	Complication and any clinical characteristic<br>s	Outcome	Score<br><br>QA type
Studies reporting transplants caused by COVID-19 infection												
								donors and abnormal in the rest				
		P1<br>M	69	HTN DM HLD	Alpha strain<br>41 days<br>Secondary bacterial infection			P1: 39F<br>GSW		P1: N/A, grade 2 PGD, 4 days ICU, 11 days hospital		
		P2<br>M	63	HTN HLD	Alpha strain, 34 days<br>6 days ARDS			P2: 40F<br>GSW		P2: bacterial sinusitis, grade 3 PGD, 13 days ICU, 28 days hospital		
		P3<br>F	47	Obesity	Alpha strain<br>89 days<br>MV<br>Secondary bacterial infection			P3: 49M<br>ICH		P3: airway complications w/ tracheostomy, grade 2 PGD, 8 days ICU, 24 days hospital		
		P4<br>M	58	DM HTN	Delta strain, 33 days<br>Secondary bacterial infection			P4: 28F<br>MVA		P4: donor specific Ab, developed anastomotic stricture requiring bronchial stenting, grade 3 PGD,		

Study	Study Type<br><br>Country	N (total)<br>Gender<br>(F%/M%)	Age<br>Mean ±SE/<br>Median<br>(IQR)<br>(years)	Comorbidities<br>(recipient)	COVID<br>Date/<br>duration<br>severity	Type of organ<br>transplant	How long<br>after COVID	Organ<br>donor	Management	Complication<br>and any<br>clinical<br>characteristic<br>s	Outcome	Score<br><br>QA type
Studies reporting transplants caused by COVID-19 infection												
										9 days ICU, 18<br>days hospital		
		P5<br>M	32	Obesity	Delta strain,<br>45 days<br>6 days ARDS<br>ECMO<br>MV			P5: 22M<br>Head<br>trauma		P5: donor<br>specific Ab,<br>thromboemb<br>olic<br>complications<br>DVT at<br>suprarenal<br>IVC, grade 2<br>PGD, 8 days<br>ICU, 28 days<br>hospital		
		P6<br>F	62	Obesity<br>HTN DM 2	Delta strain<br>156 days			P6: 37F<br>Drug<br>overdose		P6: donor<br>specific Ab,<br>Grade 3 PGD,<br>12 days ICU,<br>18 days<br>hospital<br>Died 7<br>months after<br>lung<br>transplantatio<br>n due to<br>persistent<br>infections		
Kehara et<br>al.[39]	Retrospecti<br>ve case<br>series<br><br><br>USA	20<br><br>3 F<br>17 M	Mean:<br>58 ± 12<br><br>62 (31-77)	DM<br>CAD<br>COPD<br>Asthma<br>Chronic<br>allograft	Post-COVID<br>pulmonary<br>fibrosis<br>(PCPF): 18	Double LT<br>(1 was REDO):<br>7<br><br>RLT (1 was w/<br>CABG)*: 8	163 ± 116<br>(time from<br>covid<br>diagnosis)<br><br>107 (42-368)	Age:<br>38± 14<br><br>38 (14-61)	NA	PGD requiring<br>ECMO: 3<br><br>DVT (1<br>patient with<br>major PE): 10	Post-<br>transplant<br>:<br><br>1 died<br>from	7/8<br><br>Murad et<br>al.[15]

Study	Study Type<br><br>Country	N (total)<br>Gender (F%/M%)	Age<br>Mean ±SE/ Median (IQR) (years)	Comorbidities (recipient)	COVID Date/ duration severity	Type of organ transplant	How long after COVID	Organ donor	Management	Complication and any clinical characteristics	Outcome	Score<br><br>QA type
Studies reporting transplants caused by COVID-19 infection												
		(15%/85%)		Smoking<br>Precovid lung disease<br>ILD	Post-COVID ARDS: 2	LLT (1 was w/ CABG)*: 5<br><br>Total ischemic time, min<br>324 ± 93 [325 (193–590)]<br><br>*Some patients had intraoperative CABG because of obstructive lesions		3 donors w/ diabetes<br><br>1 smoker		Intraoperative info:<br>ECMO: 8<br><br>CPB: 3<br><br>Off pump for transplant: 9	COVID associated myocarditis POD 195<br><br>3 got recurrence of COVID infection (treated and discharged)<br><br>Of 13 Single LT recipients, improvement of the native contralateral lung was observed in 5 pts: 60y/o, precovid COPD, PCPF, RLT,	

Study	Study Type<br><br>Country	N (total)<br>Gender<br>(F%/M%)	Age<br>Mean ±SE/<br>Median<br>(IQR)<br>(years)	Comorbidities<br>(recipient)	COVID<br>Date/<br>duration<br>severity	Type of organ<br>transplant	How long<br>after COVID	Organ<br>donor	Management	Complication<br>and any<br>clinical<br>characteristic<br>s	Outcome	Score<br><br>QA type
Studies reporting transplants caused by COVID-19 infection												
											10d hospitaliz ed. 64y/o, precovid ILD, PCPF, LLT, 7d<br><br>Hospitaliz ed. 67y/o, no precovid lung disease, PCPF, RLT, 25d hosp,<br><br>Died: Cardiomy opathy). 43yo, no precovid lung disease, PCPF, LLT, 14d hospitaliz ed. 63yo, precovid ILD, PCPF, LLT, 22d hosp	

Study	Study Type<br><br>Country	N (total)<br>Gender (F%/M%)	Age<br>Mean ±SE/ Median (IQR) (years)	Comorbidities (recipient)	COVID Date/ duration severity	Type of organ transplant	How long after COVID	Organ donor	Management	Complication and any clinical characteristics	Outcome	Score<br><br>QA type
Studies reporting transplants caused by COVID-19 infection												
Kiyak et al.[40]	Case Report<br><br>Turkey	1 M	35	NA	NA<br><br>Fever<br><br>Dry cough<br><br>Anosmia<br><br>Dyspnea<br><br>4 days CT showed multifocal pneumonia<br><br>36 days MV<br><br>Post-COVID-19 cholangiopathy	ldLvT	1 year	Live donor	NA	NA	All LFTs were normal within 3 months of liver transplantation	6/8<br><br>Murad et al.[15]
Koch et al.[41]	Case report<br><br>Germany	1 M	31	Crohn's disease	11/09/2020<br><br>Severe COVID-19<br><br>Day 2: Ventilation	BLT<br><br>Ischemic time for:<br><br>Right lung: 332 minutes	NA	28 YO M with ICH<br><br>Donor-recipient size matched	Uneventful<br><br>prolonged weaning phase until day 36<br><br>Temporary jejunal feeding	Day 59 post-op, underwent sternal revision for instability	Successful lung BLT<br><br>Discharged 96 days after surgery	5/8<br><br>Murad et al.[15]

Study	Study Type<br><br>Country	N (total)<br>Gender (F%/M%)	Age<br>Mean ±SE/ Median (IQR) (years)	Comorbidities (recipient)	COVID Date/ duration severity	Type of organ transplant	How long after COVID	Organ donor	Management	Complication and any clinical characteristics	Outcome	Score<br><br>QA type
Studies reporting transplants caused by COVID-19 infection												
					Day 8: Tracheostomy and ECMO<br>Day 21: **Therapy**<br>Day 56: respiratory failure with irreversible ARDS<br><br>Evaluation for lung transplantation	Left lung: 480 minutes		A lung volume reduction (middle lobe and lingula resection) was performed	tube was placed due to gastroparesis			
Lang et al.[42]	Retrospective analysis<br><br>Austria	19<br><br>(16%/84%)	56 (34–64)	NA	COVID-19 date NA<br>Time between referral and decision for listing: 14 (2–57) days<br><br>ARDS<br>Persistent pulmonary consolidations in all lobes ECMO	BLT	NA	NA	18: VV-ECMO<br><br>1: VA-ECMO: Median (range) length of 41 (2–66) days<br><br>Corticosteroids until day3<br><br>Maintenance immunosuppression:<br>FK506 (tacrolimus)-based triple	4: prolonged VA ECMO<br>1: prolonged VV ECMO	5: died post-transplant (had fully functional grafts at the time of death)<br><br>P1, 2 and 3: Died 65, 66 and 154 days after transplantation due	8/9<br><br>NOS[14]

Study	Study Type<br><br>Country	N (total)<br>Gender<br>(F%/M%)	Age<br>Mean ±SE/<br>Median<br>(IQR)<br>(years)	Comorbidities<br>(recipient)	COVID<br>Date/<br>duration<br>severity	Type of organ<br>transplant	How long<br>after COVID	Organ<br>donor	Management	Complication<br>and any<br>clinical<br>characteristic<br>s	Outcome	Score<br><br>QA type
Studies reporting transplants caused by COVID-19 infection												
									immunosuppressi<br>on regimen with:<br>Mycophenolate<br>mofetil Steroids		to<br>cholangio<br>pathic<br>liver<br>failure<br><br>P 4: Died<br>111 days<br>after<br>transplant<br>ation<br>after<br>developin<br>g multi-<br>organ<br>failure<br>due to<br>recurrent<br>infections<br><br>P 5: Died<br>147 days<br>after<br>transplant<br>ation<br>after<br>being<br>successful<br>ly<br>discharge<br>d and had<br>spontane<br>ous ICH	

Study	Study Type<br><br>Country	N (total)<br>Gender<br>(F%/M%)	Age<br>Mean ±SE/<br>Median<br>(IQR)<br>(years)	Comorbidities<br>(recipient)	COVID<br>Date/<br>duration<br>severity	Type of organ<br>transplant	How long<br>after COVID	Organ<br>donor	Management	Complication<br>and any<br>clinical<br>characteristic<br>s	Outcome	Score<br><br>QA type
Studies reporting transplants caused by COVID-19 infection												
											Early<br>post-<br>operative<br>outcome<br>was<br>worse in<br>patients<br>who<br>develope<br>d ARDS<br>due to<br>COVID-19	
Mortazavi<br>et al.[43]	Case series<br><br>USA	20<br>(40%/60%<br>)	39 (24–66)	Pregnancy<br>Ramsay hunt<br>syndrome<br>HTN<br>DVT<br>DM<br>HL<br>Drug/alcohol<br>use<br>Obesity<br>Fatty liver<br>Hypothyroidism<br>Depression<br>Ovarian cancer<br>COPD<br>Ex-smoker<br>Asthma<br>ASD	CT Chest<br>showed:<br>Consolidati<br>on with<br>ground<br>glass<br>opacificatio<br>n and<br>traction<br>bronchiecta<br>sis<br>Enlarged<br>main<br>pulmonary<br>artery<br>Air-<br>containing<br>cysts	BOLT	129 (63–711)<br>days from<br>initial COVID-<br>19	NA	NA	Vascular<br>events:<br><br>Stroke<br>Thrombosis<br>PE<br><br>Postoperative<br>infection and<br>sepsis:<br><br>Bacterial<br>Fungal by<br>*Candida sp.*<br>and<br>*Aspergillus sp*<br><br>PTX	3 died on<br>POD 13,<br>19, and<br>305<br><br>NR for<br>other<br>patients	6/8<br><br>Murad et<br>al.[15]

Study	Study Type<br><br>Country	N (total)<br>Gender<br>(F%/M%)	Age<br>Mean ±SE/<br>Median<br>(IQR)<br>(years)	Comorbidities<br>(recipient)	COVID<br>Date/<br>duration<br>severity	Type of organ transplant	How long after COVID	Organ donor	Management	Complication and any clinical characteristics	Outcome	Score<br><br>QA type
Studies reporting transplants caused by COVID-19 infection												
					Apical cystic formation<br>16: VV-ECMO<br><br>(median duration 86 (25–157) days)					Pneumomediastinum<br>Acute myelogenous leukemia (98 days after transplant)		
Ohsumi et al.[44]	Case Report<br><br>Japan	1 F	57	NA	COVID-19 related severe ARDS<br><br>Initially presented with:<br>Body weakness<br>Reduced consciousness<br>Difficulty in walking<br><br>On admission:<br>Blood oxygen saturation 78%	LDLLTx<br><br>Both grafts were flushed under ventilation with ET-Kyoto solution<br><br>Total graft ischemic time: 226 and 148 minutes (right and left lungs respectively)	NA	Right lower lobe: Patient's son<br><br>Left lower lobe: Patient's husband<br><br>Functional size matching: Calculated using FVC (79%)	2 months post-transplant: weaned from the ventilator	NA	4 months post-transplant: no complications noted	4/8<br><br>Murad et al.[15]

Study	Study Type<br><br>Country	N (total)<br>Gender (F%/M%)	Age<br>Mean ±SE/ Median (IQR) (years)	Comorbidities (recipient)	COVID Date/ duration severity	Type of organ transplant	How long after COVID	Organ donor	Management	Complication and any clinical characteristics	Outcome	Score<br><br>QA type
Studies reporting transplants caused by COVID-19 infection												
					Intubated<br>MV<br>PaO2 57 mm Hg<br>ECMO							
Umemura et al.[45]	Case report<br><br>Japan	1 F	50	NA	NA<br><br>COVID-19-related respiratory failure	LDLLTx	104 days after onset of COVID-19	NA	Immunosuppressive regimen:<br>Tacrolimus<br>Mycophenolate mofetil Cortico-steroids<br>PPI<br>ITCZ	Nausea and vomiting after taking ITCZ	No invasive fungal infections observed in first 4 months after transplant<br><br>POD 117, the patient was CMV positive and was transferred to rehabilitation on POD 131<br><br>No gastric ulcers or hyperglycemia	5/8<br><br>Murad et al.[15]

Study	Study Type<br><br>Country	N (total)<br>Gender (F%/M%)	Age<br>Mean ±SE/ Median (IQR) (years)	Comorbidities (recipient)	COVID Date/ duration severity	Type of organ transplant	How long after COVID	Organ donor	Management	Complication and any clinical characteristics	Outcome	Score<br><br>QA type
Studies reporting transplants caused by COVID-19 infection												
Uhl et al.[46]	Case series<br><br>USA	12<br><br>NA	NA	NA	IPF<br>Post-COVID fibrosis	LT	NA	NA	NA	NA	NA	3/8<br><br>Murad et al.[15]
Lee et al.[47]	Case report<br><br>USA	1 M	64	HTN<br>HLD<br>IDDM<br>Prostate cancer	Severe Pneumonia<br>Acute respiratory failure<br>Intubated<br>ICU<br>DVT<br><br>Developed:<br>Large iliopsoas Hematoma<br>Candidemia<br>Sclerosing cholangitis leading to end-stage liver disease<br>Diffuse hepatic injury and fibrosis	LvT	259 after COVID-19 diagnosis	Deceased donor	NA	NA	POD 1, NGT removed<br><br>Liver US demonstrated patent vessels<br><br>POD 4, discharged with AST 107, ALT 215, TBIL 3.7mg/dl	6/8<br><br>Murad et al.[15]
Magnusson et al.[48]	Case Report<br><br>Sweden	1 M	55	NA	NA<br><br>Initial presentation:	BLT	138<br><br>(after ECMO support)	NA	POD 2: patient went for surgery for removal of clots	Moderate bleeding	Successful BLT<br><br>No signs of	5/8<br><br>Murad et al.[15]

Study	Study Type<br><br>Country	N (total)<br>Gender<br>(F%/M%)	Age<br>Mean ±SE/<br>Median<br>(IQR)<br>(years)	Comorbidities<br>(recipient)	COVID<br>Date/<br>duration<br>severity	Type of organ<br>transplant	How long<br>after COVID	Organ<br>donor	Management	Complication<br>and any<br>clinical<br>characteristic<br>s	Outcome	Score<br><br>QA type
Studies reporting transplants caused by COVID-19 infection												
					Fever<br>Increased<br>respiratory<br>rate<br>Arterial<br>partial<br>pressure of<br>O2 of 5.9<br>kPa despite<br>noninvasive<br>high flow<br>O2<br><br>MV<br>ECMO<br>Irreversible<br>lung<br>Required LT				PGD graded as 1<br>at 24 hours, 1 at<br>48 hours, and 0 at<br>72 hours<br><br>POD 14: Weaned<br>from the<br>ventilator<br><br>POD 20:<br>Discharged from<br>the ICU<br><br>POD 34:<br>Discharged to<br>rehabilitation	Transient<br>atrial<br>fibrillation	infection<br>or<br>transplant<br>rejection<br><br>Donor-<br>specific<br>Ab were<br>negative<br><br>Discharge<br>d on POD<br>34	
Cooper et<br>al.[49]	Retrospecti<br>ve case<br>series<br><br>Israel	2 M										5/8<br><br>Murad et<br>al.[15]
		P1	3 months	Late preterm<br>(36 wks)<br><br>Low<br>birthweight<br>(2300 g)	Feb 2021<br>Severe<br>Progressive<br>jaundice<br><br>Coagulopat<br>hy<br>Acute liver<br>failure at	Live left<br>lateral liver<br>transplant on<br>day 32 post<br>covid<br>diagnosis<br><br>Histology of<br>explant	Transplant on<br>day 32 post<br>covid<br>diagnosis	Father	NA	NA	Recovere<br>d well and<br>was<br>continuin<br>g<br>ambulato<br>ry follow<br>up at time<br>of study	

Study	Study Type<br><br>Country	N (total)<br>Gender<br>(F%/M%)	Age<br>Mean ±SE/<br>Median<br>(IQR)<br>(years)	Comorbidities<br>(recipient)	COVID<br>Date/<br>duration<br>severity	Type of organ<br>transplant	How long<br>after COVID	Organ<br>donor	Management	Complication<br>and any<br>clinical<br>characteristic<br>s	Outcome	Score<br><br>QA type
Studies reporting transplants caused by COVID-19 infection												
					day 21 post<br>covid<br>diagnosis<br><br>Deteriorate<br>d and<br>became<br>encephalop<br>athic	showed<br>pericentral<br>and<br>panlobular<br>necrosis<br><br>Negative for<br>EBV, CMV,<br>HSV, and<br>SARS-COV2						
		P2	5 months	Hypospadias<br>GERD	May 2021,<br><br>Severe<br><br>Jaundice<br>Hepatomeg<br>aly<br>Progressed<br>to<br>secondary<br>HLH with<br>cytopenia<br>Deterioratio<br>n and<br>encephalop<br>athy<br><br>LvT was<br>performed	Lvt<br><br>Histology of<br>explant<br>showed<br>massive<br>panlobular<br>necrosis with<br>areas of<br>predominantl<br>y pericentral<br>necrosis<br><br>Negative for<br>adenovirus,<br>EBV, CMV,<br>HSV, and<br>SARS-CoV-2	NA	Live left<br>lateral liver<br>transplant<br><br>Mother	NA	Post<br>operatively +<br>for CMV and<br>adenovirus<br><br>Treated with<br>ganciclovir<br>and cidofovir	HLH<br>resolved<br>spontane<br>ously<br><br>Negative<br>for CMV,<br>adenoviru<br>s, and<br>COVID-19	
Studies reporting transplants post-COVID-19 infection (recipient had COVID-19 but organ damage was caused by other factors)												

Study	Study Type<br><br>Country	N (total)<br>Gender<br>(F%/M%)	Age<br>Mean ±SE/<br>Median<br>(IQR)<br>(years)	Comorbidities<br>(recipient)	COVID<br>Date/<br>duration<br>severity	Type of organ<br>transplant	How long<br>after COVID	Organ<br>donor	Management	Complication<br>and any<br>clinical<br>characteristic<br>s	Outcome	Score<br><br>QA type
Studies reporting transplants caused by COVID-19 infection												
Akbulut et al.[50]	Observation al retrospectiv e study<br><br>Case series<br><br>Turkey	35<br><br>(34.3%/65 .7%)	50<br>(95% CI 43-54)	DM<br>CAD	NA<br><br>Hospitalized : 8<br><br>Intubated in ICU: 1<br><br>Fever: 14<br><br>Loss of appetite: 13<br><br>Cough and loss of tase: 9<br><br>Dyspnea: 5<br><br>Headache: 10<br><br>Low back pain: 6<br>Fatigue: 20<br><br>Myalgia and loss of smell: 10	LvT<br><br>Liver graft type:<br><br>Right: 27<br>Left: 4<br>Left lateral: 4<br><br>Median graft-to-recipient weight ratio: 1.01 (95% CI 0.96-1.28)<br><br>Median implanted liver graft weight: 755 g (95% CI 690-780)	37 days<br>(95% CI 26-54)	Living donor: 3<br><br>Deceased donor: 2	NA	Acute rejection: 3<br><br>Chronic rejection: 1<br><br>Early hepatic artery thrombosis: 4<br><br>Death due to:<br>Cardiac problems: 4<br>Complications associated with mesenteric ischemia: 1<br><br>No death secondary to complications associated with COVID-19	Post transplant rejection: 4 at a median 25 days after LT<br><br>Dead: 5 within median 23 days after LT	5/8<br><br>Murad et al.[15]
Antal et al.[51]	Case report<br><br>Romania	1 M	47	ESCK	09/02/2022	KT	2 weeks after COVID-19	Cadaver donor	Low CO and normal SVV, PPV and SVR after	No surgical complications	Discharge d from hospital	4/8

Study	Study Type<br><br>Country	N (total)<br>Gender<br>(F%/M%)	Age<br>Mean ±SE/<br>Median<br>(IQR)<br>(years)	Comorbidities (recipient)	COVID<br>Date/<br>duration<br>severity	Type of organ transplant	How long after COVID	Organ donor	Management	Complication and any clinical characteristics	Outcome	Score<br><br>QA type
Studies reporting transplants caused by COVID-19 infection												
				Hepatitis C cryoglobulinemia<br><br>Nephropathy<br><br>Chronic rejection of a previous ldKT in 2002<br><br>Hepatitis B virus<br><br>Pancytopenia<br><br>Secondary HTN<br><br>Hypothyroidism	Low grade fever for 24 hours and fatigability treated with 5 days of molnupiravir		with minimal symptoms		induction of anesthesia<br><br>Low dose of dopamine to allow organ perfusion and prevent ischemic complications<br><br>Maintenance immunosuppressive therapy: Mycophenolic acid Tacrolimus Methylprednisone<br><br>Prophylaxis: Trimethoprim-sulfamethoxazole Valganciclovir in renal doses	Cold ischemia time <12h<br><br>Transplant duration 120 min<br><br>Delayed graft function immediately after transplantation (required 1 hemodialysis session on POD 5)<br><br>Urinary output increased from 0.5 ml/kg/h immediately after transplant to 3 ml/kg/h on POD 3<br><br>Creatinine level decreased up to 194.5 µmol/L	on POD 28	Murad et al.[15]

Study	Study Type<br><br>Country	N (total)<br>Gender<br>(F%/M%)	Age<br>Mean ±SE/<br>Median<br>(IQR)<br>(years)	Comorbidities<br>(recipient)	COVID<br>Date/<br>duration<br>severity	Type of organ<br>transplant	How long<br>after COVID	Organ<br>donor	Management	Complication<br>and any<br>clinical<br>characteristics	Outcome	Score<br><br>QA type
Studies reporting transplants caused by COVID-19 infection												
										Developed thrombocytopenia and leukopenia on POD 6<br><br>Developed mild pancreatitis and incidental 2 mm calculus of the gallbladder two weeks after transplantation		
Singh et al.[52]	Case report<br><br>USA	1 F	66	ESRD<br><br>DM	04/19/2020<br><br>3 months prior to transplantation<br><br>Fever and cough<br><br>Recovered in self-quarantine at home	Simultaneous pancreas and kidney transplant	3 months	NA	POD 3: Reduced doses of rabbit anti-thymocyte globulin<br><br>Intravenous methylprednisolone taper<br><br>POD5: Discharge on mycophenolate mofetil	No complications	Asymptomatic with no signs of repeat COVID-19 infection 7 weeks after transplant<br><br>Discharged on POD	6/8<br><br>Murad et al.[15]

Study	Study Type<br><br>Country	N (total)<br>Gender (F%/M%)	Age<br>Mean ±SE/ Median (IQR) (years)	Comorbidities (recipient)	COVID Date/ duration severity	Type of organ transplant	How long after COVID	Organ donor	Management	Complication and any clinical characteristic s	Outcome	Score<br><br>QA type
Studies reporting transplants caused by COVID-19 infection												
					Negative PCR recorded on July 2 2020				Extended-release tacrolimus<br><br>Oral prednisone taper		5	
Sherwood et al.[53]	Case report<br><br>Canada	1 M	30	IgA Nephropathy	COVID-19 positive POD2<br><br>Cough<br>Dyspnea<br>Fever	ldKT	COVID-19 negative before and on the day of transplant<br><br>Tested positive on POD2	Negative donor-specific antibody<br><br>Donor tested negative before the operation	Basiliximab on day 0 and 4<br><br>Maintenance:<br>Prednisone<br>Tacrolimus<br>Mycophenolate mofetil	COVID-19 symptoms fully resolved after 1 month<br><br>Borderline acute cellular rejection at week 10 (treated with pulse methylpredni solone)	Discharge d on POD7<br><br>Symptom s fully resolved by POD 30	6/8<br><br>Murad et al.[15]

Study	Study Type<br><br>Country	N (total)<br>Gender<br>(F%/M%)	Age<br>Mean ±SE/<br>Median<br>(IQR)<br>(years)	Comorbidities<br>(recipient)	COVID<br>Date/<br>duration<br>severity	Type of organ<br>transplant	How long<br>after COVID	Organ<br>donor	Management	Complication<br>and any<br>clinical<br>characteristic<br>s	Outcome	Score<br><br>QA type
Studies reporting transplants caused by COVID-19 infection												
										CMV viremia<br>between<br>weeks 14 and<br>20 post-<br>transplant		
Kute et al.[54]	Retrospecti<br>ve cohort<br>study<br><br>India	38<br><br>32 F<br>6 M	38.5 (31.25-<br>47.5)	HTN<br><br>DM	Study was<br>done<br>between<br>April 2020<br>to<br>December<br>31, 2020<br><br>Exact date<br>of COVID-19<br>infection:<br>NA<br><br>Fever: 31<br><br>Cough: 26<br><br>Difficulty<br>breathing: 7<br><br>Anosmia: 9<br>Nausea/vo<br>miting: 5<br><br>Fatigue/mal<br>aise: 14	KT<br><br>Most<br>common<br>incompatibilit<br>y:<br><br>A to O: 14<br><br>B to O: 10<br><br>A to B, B to A,<br>and AB to A: 4<br>(5.2%) pairs<br>each<br><br>AB to B: 2<br>(2.6%)	Days from -ve<br>PCR to<br>surgery 118<br>(55-168)	All donors<br>were<br>females	Treatment<br>regimen used:<br><br>Remdesivir<br><br>Anticoagulation<br><br>Steroids<br><br>Ivermectin<br><br>Doxycycline<br><br>Azithromycin	1 death at 6<br>months of<br>transplant<br>with fungal<br>pyelonephriti<br>s which<br>suffered graft<br>loss during<br>treatment	Acute<br>rejection:<br>13.1%<br><br>Graft loss:<br>2.6%<br><br>Mortality:<br>2.6%	7/9<br><br>NOS[14]

Study	Study Type<br><br>Country	N (total)<br>Gender<br>(F%/M%)	Age<br>Mean ±SE/<br>Median<br>(IQR)<br>(years)	Comorbidities<br>(recipient)	COVID<br>Date/<br>duration<br>severity	Type of organ<br>transplant	How long<br>after COVID	Organ<br>donor	Management	Complication<br>and any<br>clinical<br>characteristic<br>s	Outcome	Score<br><br>QA type
Studies reporting transplants caused by COVID-19 infection												
					Diarrhea: 1<br><br>Oxygen<br>requiremen<br>ts:<br>Did no<br>require: 19<br><br>Low flow: 8<br><br>BiPAP: 1							
Kute et al.[55]	Retrospecti<br>ve cohort<br>study<br><br>India	75<br><br>8 F<br><br>67 M	39.4 ± 12,<br>40 (7–62)	HTN<br><br>DM<br><br>Obesity<br><br>CAD<br><br>Anemia<br><br>hypothyroid	COVID-19<br>date/durati<br>on- NA<br><br>Asymptoma<br>tic: 17<br><br>Mild: 36<br><br>Moderate:<br>15<br><br>Severe: 7<br><br>ICU: 7<br><br>Ventilation:<br>1	KT<br><br>ABO-<br>incompatible:<br>12<br><br>ABO-<br>compatible:<br>63<br>(with<br>negative<br>complement-<br>dependent<br>cytotoxicity<br>and flow<br>crossmatch<br>pretransplant<br>)	Median<br>duration<br>between<br>SARS-CoV-2<br>PCR positivity<br>to transplant<br>was 60 days	Age: 47 ±<br>10, 47 (29–<br>72)<br><br>Gender:<br><br>52 F<br><br>23 M<br><br>No<br>comorbiditi<br>es<br><br>COVID- 19<br>negative: 59	Thymoglobulin:<br>42<br>Basiliximab: 24	Acute<br>rejection: 5<br><br>Antibody-<br>mediated<br>rejection: 2<br><br>T cell–<br>mediated: 3<br><br>Acute tubular<br>necrosis in<br>the first<br>month: 3<br><br>All rejections<br>responded<br>well to<br>methylpredni	100%<br>patient<br>and graft<br>survival	8/9<br><br>NOS[14]

Study	Study Type<br><br>Country	N (total)<br>Gender (F%/M%)	Age<br>Mean ±SE/ Median (IQR) (years)	Comorbidities (recipient)	COVID Date/ duration severity	Type of organ transplant	How long after COVID	Organ donor	Management	Complication and any clinical characteristics	Outcome	Score<br><br>QA type
Studies reporting transplants caused by COVID-19 infection												
					All recipients were COVID-19 negative at the time of transplant			Hx of COVID-19: 16<br><br>Asymptomatic: 13; Mild: 3		solone 500mg pulses × 3 d with an improvement in their renal function.<br><br>1 developed posttransplant diabetes<br><br>1 had urinary tract infection		
Jacob et al.[56]	Case Report<br><br>Australia	1 F	39	NA	5 days before admission<br><br>Acute COVID-19<br><br>Fever<br>Cough<br>Coryzal symptoms for 5 days<br><br>Admitted as a case of acute liver failure	LvT	This case of liver failure is during acute COVID infection and NOT post COVID	Brain death allograft	Tenofovir disoproxil fumarate (TDF)<br><br>Developed grade 3/4 encephalopathy requiring intubation and continuous renal replacement therapy<br><br>On day 4 of admission, induction immunosuppressi	Before transplantation<br><br>Grade 3/4 encephalopathy requiring intubation and continuous renal replacement therapy<br><br>After transplantation	Discharged on POD 14<br><br>Recovered by POD 30 with normal liver function and negative SARS-CoV-2 PCR on POD 28	5/8<br><br>Murad et al.[15]

Study	Study Type<br><br>Country	N (total)<br>Gender<br>(F%/M%)	Age<br>Mean ±SE/<br>Median<br>(IQR)<br>(years)	Comorbidities<br>(recipient)	COVID<br>Date/<br>duration<br>severity	Type of organ<br>transplant	How long<br>after COVID	Organ<br>donor	Management	Complication<br>and any<br>clinical<br>characteristic<br>s	Outcome	Score<br><br>QA type
Studies reporting transplants caused by COVID-19 infection												
									on was given with<br>ethylprednisone<br>& basilixmab	Maintenance<br>immunosuppr<br>ession:<br>Prednisone<br>Tacrolimus<br>TDF		
Okubo et al.[57]	Case Report<br><br>USA	1 M	65	DM<br><br>Nonalcoholic<br>steatohepatitis<br>cirrhosis<br>complicated by:<br><br>AscitesNon<br>bleeding<br>esophageal<br>varices<br>Hepatocellular<br>carcinoma<br><br>Underwent<br>OLvT in March<br>2020<br><br>complicated by<br>cholestatic graft<br>failure<br><br>Relisted for<br>transplant<br>8/2020	08/13/2020<br>, PCR<br>positive<br>while being<br>evaluated<br>for a<br>potential<br>organ offer<br><br>Asymptoma<br>tic<br><br>Chest X-ray<br>and CT<br>without IV<br>contrast<br>showed<br>pulmonary<br>manifestati<br>ons of<br>COVID-19	OlvT	Patient had<br>persistent<br>PCR positivity<br>for 40 days<br>before<br>retransplant<br><br>(likely in the<br>setting of<br>immunosuppr<br>ession)<br><br>On<br>September 9,<br>2020, SARS-<br>CoV-2 Ab<br>were<br>detected<br><br>Due to lack of<br>COVID-19<br>symptoms,<br>patient was<br>reactivated<br>for transplant	NA	Postoperative<br>course for acute<br>blood loss anemia<br>(required a return<br>to the operating<br>room for a<br>washout on POD<br>1<br><br>Weaned from MV<br>on POD 2 without<br>any respiratory<br>complication<br>Mycophenolate<br>mofetil<br>Tacrolimus<br>Standard steroid<br>taper	None	Successful<br>transplant<br>with d no<br>viral<br>transmissi<br>on<br><br>Discharge<br>d with<br>home<br>health<br>services	6/8<br><br>Murad et<br>al.[15]

Study	Study Type<br><br>Country	N (total)<br>Gender<br>(F%/M%)	Age<br>Mean ±SE/<br>Median<br>(IQR)<br>(years)	Comorbidities<br>(recipient)	COVID<br>Date/<br>duration<br>severity	Type of organ<br>transplant	How long<br>after COVID	Organ<br>donor	Management	Complication<br>and any<br>clinical<br>characteristic<br>s	Outcome	Score<br><br>QA type
Studies reporting transplants caused by COVID-19 infection												
							Lvt was on<br>09/27/2020					
Juric et al.[58]	Retrospecti<br>ve<br>case series<br><br>Croatia	9<br><br>5 M<br>4 F	40.8<br>(18-71)	NA	Asymptoma<br>tic<br>infection: 2<br><br>Mild: 5<br><br>Bilateral<br>SARS-COV-2<br>pneumonia:<br>2<br><br>P1:<br>asymptoma<br>tic<br>P2: mild<br>P3: mild<br>P4: mild<br>P5:<br>pneumonia<br>P6: mild<br>P7:<br>pneumonia<br>P8:<br>asymptoma<br>tic<br>P9: mild	Kidney<br><br>8 deceased<br>donors	Avg time to<br>transplant: 8<br>months<br><br>Range: 3-13<br>months<br><br><br>P1: 3<br>P2:9<br>P3:7<br>P4:3<br>P5:9<br>P6:5<br>P7:11<br>P8:12<br>P9:13<br>(^months)	NA	Two patients w/<br>intensive<br>immunosuppressi<br>ve therapy:<br>Immunoadsorptio<br>n<br>Steroid pulses<br><br>Others received<br>standard<br>immunosuppressi<br>ve therapy:<br>Basiliximab<br>induction<br><br>Tacrolimus or<br>mTOR- inhibitor<br><br>Mycophenolate<br>mofetil<br>Steroids	2 pts who<br>were highly<br>immunized<br>were treated<br>with<br>immunoadsor<br>ption	NA	2/8<br><br>Murad et<br>al.[15]
Studies reporting transplants when the donor had COVID-19												

Study	Study Type<br><br>Country	N (total)<br>Gender<br>(F%/M%)	Age<br>Mean ±SE/<br>Median<br>(IQR)<br>(years)	Comorbidities<br>(recipient)	COVID<br>Date/<br>duration<br>severity	Type of organ transplant	How long after COVID	Organ donor	Management	Complication and any clinical characteristics	Outcome	Score<br><br>QA type
Studies reporting transplants caused by COVID-19 infection												
Sanchez-Vivaldi et al.[59]	Retrospective case series<br><br>USA	9<br>5 F<br>4 M<br><br>(55.6%/44.4%)	45.0 (32.0–54.0)	CKD<br>ESRD	NA	KT	NA	13 SARS-COV positive deceased donors<br><br>28.6% F<br>71.4% M<br><br>2 brain death<br><br>5 cardiac deaths	Most recipients received Tacrolimus Mycophenolate Steroids	No complications after transplant	No major complications, graft loss, vessel thrombosis or donor-derived COVID-19 infections<br><br>2 (22.2%) had DGF<br><br>Median hospital stay: 4 days<br><br>All recipients had satisfactory allograft function (median creatinine of 1.51 mg/dl) at 30-day follow-up	6/8<br><br>Murad et al.[15]

Study	Study Type<br><br>Country	N (total)<br>Gender<br>(F%/M%)	Age<br>Mean ±SE/<br>Median<br>(IQR)<br>(years)	Comorbidities<br>(recipient)	COVID<br>Date/<br>duration<br>severity	Type of organ<br>transplant	How long<br>after COVID	Organ<br>donor	Management	Complication<br>and any<br>clinical<br>characteristics	Outcome	Score<br><br>QA type
Studies reporting transplants caused by COVID-19 infection												
Nguyen et al.[60]	Case Report<br><br>USA	1 M	24	Crohn's disease<br><br>PSC	Confirmed SARS-CoV-2 infection 3 d following donation<br><br>Donor contracted the virus shortly before surgery given the timing of symptoms	Right hepatic lobe	NA	Live donor<br><br>Donor was advised to isolate 7 days before surgery<br><br>Tested negative for SARS-CoV-2 day 3 preoperative	Postoperative immunosuppression: Tacrolimus, Mycophenolate mofetil<br>Tapering doses of methylprednisolone<br><br>Negative for SARS-CoV-2 on POD 4 and POD 5<br><br>Approved for prophylactic treatment with 1 unit of CCP on POD 4	No viral transmission<br><br>Both donor and recipient had excellent postoperative outcomes		4/8<br><br>Murad et al.[15]

Ab: Antibodies, **AKI**: Acute Kidney Failure, ALT: alanine transaminase, **ANA**: Antinuclear Antibody, **APOLvT**: Auxiliary Partial Orthotopic Liver Transplantation, **Approx**.: Approximately, **ARDS**: Acute Respiratory Distress Syndrome, **AS**: Ankylosing Spondylitis, **ASD**: Arterial Septal Defect, **AST**: aspartate aminotransferase, **BAL**: Bronchoalveolar Lavage, **BLT**: Bilateral Lung Transplant, **BOLT**: Bilateral Orthotopic Lung Transplantation, **CABG**: Coronary Artery Bypass Graft, **CAD**: Coronary Artery Disease, **CI**: Cerebral Ischemia, **CMV**: Cytomegalovirus, **CNS**: Central Nervous System, **COPD**: Chronic Obstructive Lung Disease, **COVID**-19: Coronavirus Disease 2019, **CVA**: Cerebral Vascular Accident, **DB**: Direct bilirubin, **ddBLT**: Deceased Donor Bilateral Lung Transplant, **ddLvT**: Deceased Donor Liver Transplant, **DM**: Diabetes Mellitus, **DNA**: Deoxyribonucleic Acid, **DVT**: Deep Vein Thrombosis, **ECMO**: Extracorporeal Membrane Oxygenation, **ERCP**: Endoscopic Retrograde Cholangiopancreatography, **ESRD**: End Stage Renal Disease, **ESLD**: End-Stage Lung Disease, **FEV**: Forced Expiratory Volume, **Fio2**: Fraction Of Inspired Oxygen, **FVC**: Forced Vital Capacity , **GERD**: Gastroesophageal Reflux Disease, **GI**: Gastrointestinal, **GN**: Gram-Negative, **GSW**: Gunshot Wound , **HCV**: Hepatitis C Virus, **HLD**: Hyperlipidemia, **HLH**: Hemophagocytic Lymphohistiocytosis, **HoS**: History Of Smoking, **HT**: Heart Transplant, **HTN**: Hypertension, **HTX**: Hemothorax, **Hx**: History, **ICH**: Intracerebral Hemorrhage, **ICU**: Intensive Care Unit, **IgG**: Immunoglobulin G, **ILD:** Interstitial Lung Disease, **IPF**: Idiopathic pulmonary Fibrosis, **IQR**: Interquartile Range, **ITCZ**: Itraconazole, **IVC**: Inferior Vena Cava, **KPC XDR**: Extensively Drug-Resistant Carbapenem-Producing Klebsiella pneumonia, **KT**: Kidney Transplant, **ldKT**: Living Donor Kidney Transplant, **ldLLT**: Living-Donor Lobar Lung Transplantation, **ldLT**: Living Donor

Lung Transplant, **ldLvT**: Living Donor Liver Transplant, **LFT**: Liver Function Tests, **LMWH**: Low-Molecular-Weight Heparin, **LT**: Lung Transplant, **Lvt**: Liver Transplant, **MDA5**: Anti-Melanoma Differentiation-Associated Gene 5, **MDR**: Multidrug Resistant, **MRCP**: Magnetic Resonance Cholangiopancreatography, **MSSA**: Methicilin-Susceptible Staphylococcus Aureus, **MVA**: Motor Vehicle Accident, **MV**: Mechanical Ventilation, **NA**: Not Applicable/Mentioned, **NGT**: Nasogastric Tube, **O2 Sat.**: Oxygen Saturation, **OLvT**: Orthotopic Liver Transplant, **OSA**: Obstructive Sleep Apnea, **Pao2**: Partial Pressure Of Oxygen In The Arterial Blood, **PCPF**: Post-COVID Pulmonary Fibrosis, **PCR**: Polymerase Chain Reaction, **PE**: Pulmonary Embolism, **PGD**: Primary Graft Dysfunction, **PLS**: Passenger Lymphocyte Syndrome, **POD**: Postoperative Day, **PTX**: Pneumothorax, **RT-qPCR**: Reverse Transcription-Quantitative Polymerase Chain Reaction, **RTA**: Road Traffic Accident, **SARS-Cov-2**: Severe Acute Respiratory Syndrome Coronavirus 2, **SOB**: Shortness Of Breath, **SSC**: Secondary Sclerosing Cholangitis, **TB**: Total bilirubin, **TBI**: Traumatic Brain Injury, **Tx**: Transplant/Transplantation, **UC**: Ulcerative Colitis, **UFH**: Unfractionated Heparin, **VV-ECMO**: Venovenous Extracorporeal Membrane Oxygenation, **VA-ECMO**: Venoarterial Extracorporeal Membrane Oxygenation, **YO**: Year-Old.
